# Comparative Exoproteome Analysis of *Streptococcus suis* Human Isolates

**DOI:** 10.3390/microorganisms9061287

**Published:** 2021-06-12

**Authors:** Esther Prados de la Torre, Antonio Rodríguez-Franco, Manuel J. Rodríguez-Ortega

**Affiliations:** Departamento de Bioquímica y Biología Molecular, Universidad de Córdoba, and Campus de Excelencia Internacional CeiA3, 14071 Córdoba, Spain; b52prtoe@uco.es (E.P.d.l.T.); arfranco@uco.es (A.R.-F.)

**Keywords:** *Streptococcus suis*, zoonosis, human infection, proteomics, secreted proteins, vaccine candidates, moonlighting proteins

## Abstract

The swine pathogen *Streptococcus suis* is a Gram-positive bacterium which causes infections in pigs, with an impact in animal health and in the livestock industry, and it is also an important zoonotic agent. During the infection process, surface and secreted proteins are essential in the interaction between microorganisms and their hosts. Here, we report a comparative proteomic analysis of the proteins released to the extracellular milieu in six human clinical isolates belonging to the highly prevalent and virulent serotype 2. The total secreted content was precipitated and analyzed by GeLC-MS/MS. In the six strains, 144 proteins assigned to each of the categories of extracellular or surface proteins were identified, as well as 680 predicted cytoplasmic proteins, many of which are putative moonlighting proteins. Of the nine predicted signal peptide-I secreted proteins, seven had relevant antigenic potential when they were analyzed through bioinformatic analysis. This is the first work comparing the exoproteome fraction of several human isolates of this important pathogen.

## 1. Introduction

The Gram-positive bacterium *Streptococcus suis* causes a wide variety of diseases in pigs worldwide, ranging from local infections in joints and organs to fatal invasive disease [1]. This pathogen is responsible for important economic losses in the swine industry [2]. In addition to its impact on animal health and the livestock economy, *S. suis* is zoonotic, i.e., it infects humans who are in close contact with the diseased animals or related by-products [3]. In some South-East Asian countries like Thailand and Vietnam, infections caused by *S. suis* are responsible for a high number of meningitis cases in adults [3,4]. Many cases of infections in humans have been described throughout all continents, and they are mainly linked to the major *S. suis* serotype 2 (SS2) [5]. However, the most serious outbreaks took place in China in 1998 and 2005, with high mortality rates that caused tens of deaths [6].

Many efforts have been invested in developing an effective vaccine against this microorganism, but there is no licensed protective formula available yet. In recent times, interest has been placed in protein subunit-based vaccines as, if they are based on conserved protein sequences, they are expected to confer broad protection against all or most of the 35 capsular serotypes described so far [7]. Surface proteins are *a priori* the most promising protein candidates to that end, as they have the highest chances to raise the protective immune responses in the host [8]. Proteomics has been used to determine the “pan-surfomic” repertoire of either pig [9] or *S. suis* human clinical isolates [10] using the “shaving” approach [11,12]. In addition to surface-attached proteins, those that are secreted extracellularly are also of interest, as they also play significant roles in the host–pathogen interaction. In actuality, some works have reported that the secreted proteins of *S. suis* clinical isolates from pigs are recognized by the sera of infected animals [13,14,15], indicating that this group of proteins can also contribute to the provision of candidates for vaccines or diagnostic purposes. However, there is a lack of studies on extracellularly released proteins from *S. suis* human isolates. Here, we present the first report comparing the total exoproteome fractions in a collection of human clinical isolates from this pathogen.

## 2. Materials and Methods

### 2.1. Bacterial Strains, Culture Conditions and the Preparation of Exoproteomes

Six SS2 strains isolated from humans with meningitis from six different Spanish provinces were used for this study. Three of them were isolated from blood and the other three from cerebrospinal fluid [10]. They were kept at −80 °C as 15% glycerinates. For cultivation, they were first grown on Columbia agar blood-based plates containing 6% (*v*/*v*) sheep blood, and then grown in Todd-Hewitt broth at 37 °C in a 5% CO_2_ atmosphere until the mid-exponential phase (OD_600_ = 0.25). The exoproteomes were obtained after pelleting the bacteria by centrifugation at 3500× *g*, and then the supernatant was centrifuged again at 10,000× *g*. Then, this second supernatant containing the secreted proteins and other structures (i.e., the exoproteome) was precipitated with trichloroacetic acid. The resulting pellet was dissolved in 100 µL Laemmli buffer. The protein content was determined using the Bradford method [16].

### 2.2. Sample Preparation for Proteomic Analysis

An aliquot of 30 µg of each sample was run in 12% SDS-PAGE in order to obtain a general protein profile after Coomassie Brilliant Blue R250 staining. A second 12% SDS-PAGE was run allowing the samples to enter only the upper part of the resolving gel, and gel slices containing the whole sample proteins were then cut from that part to be destained, cleaned, digested with 40 µL trypsin solution (10 ng/µL) and desalted using ZipTip® columns (Merck-Millipore, Burlington, MA, USA) for further analysis by LC-MS/MS (GeLC-MS/MS).

### 2.3. LC-MS/MS Analysis and Protein Identification through a Database Search

The peptide separation by liquid chromatography, mass spectrometry analysis and protein identification were performed as previously described by our group [10]. Briefly, the peptides (5 µL of each exoproteome digested sample) were separated in a Dionex Ultimate 3000 nano UPLC (Thermo Scientific, San Jose, CA) equipped with a reverse phase C18 75 μm × 50 cm Acclaim Pepmap column (Thermo Scientific, San Jose, CA, USA) at 300 nL/min and 40 °C for a total run time of 85 min. Previously, the peptide mixtures were concentrated and cleaned up on a precolumn cartridge using 2% ACN/0.05% formic acid for 5 min, with a flow of 5 µL/min. The chromatographic separation was carried out using, as the mobile phases, Solution A (0.1% formic acid) and Solution B (80% ACN, 0.1% formic acid).

The peptide positive ions eluted from the column were ionized using a nano-electrospray ionization source and analyzed in positive mode on a trihybrid Thermo Orbitrap Fusion (Thermo Scientific, San Jose, CA, USA) mass spectrometer. The raw MS/MS data were processed with Proteome Discoverer 2.1 (Thermo Scientific, San Jose, CA, USA), and the MS/MS spectra were searched using SEQUEST v.27 against a local database that contained all of the *Streptococcus suis* BM407 proteins predicted from the genome (downloaded from UniProt on 17 July 2019 [17]). The validation of the peptide spectral matches (PSM) was carried out with Percolator at 1% FDR, based on q-values.

### 2.4. Computational Analysis of the Protein Sequences

The subcellular localization of the proteins was assigned via the online algorithm LocateP v2 [18], and was contrasted with other web-based algorithms: TMHMM 2.0 [19], SignalP 5.0 [20] and LipoP 1.0 [21]. The prediction of the antigenicity of the protein sequences was performed using VaxiJen 2.0 [22].

### 2.5. Data and Statistical Analysis

Three biological replicates were created for each exoproteome, each one from one independent culture. The proteins were considered to be identified in a sample if they were found in at least two out of the three biological replicates for such a sample. In the case that only one value was missing, the average of the two other values was calculated for such a missing value. Those proteins identified in none or only one replicate were discarded from the overall count of the identified proteins. The quantitative analysis was carried out as described [10]: the proteins were quantified from the precursor ion peak areas after normalization using Proteome Discoverer 2.1 (Thermo Scientific, San Jose, CA, USA). The means and standard deviations were calculated using an Excel spreadsheet (Microsoft Excel 2011 v14.0.0 for Mac). The values were z-scored prior to the principal component and clustering analyses. The principal component analysis was performed with the R package FactoMineR. The factoextra package was used to represent these analyses, and the pheatmap package was used to cluster the data and represent the corresponding heatmaps. The non-detected proteins in the samples were assigned a 0 value in order to avoid the processing of NA (not available) data.

## 3. Results and Discussion

A first glance at the total exoproteomes of each clinical isolate obtained through SDS-PAGE confirmed that the samples contained enough bands for further GeLC-MS/MS analysis (Figure S1). The precipitated material yielded a high number of protein species. The band pattern was quite similar in four out of the six isolates (i.e., 41/14, 857/06, 1299/06, and 117/12), showing that the other two strains (1086/11 and 34/11) had a slightly different protein profile.

Samples of the total protein content were prepared for proteomic analysis using a GeLC-MS/MS approach. Three biological replicates of each total exoproteome were loaded onto an SDS-PAGE, and a unique band was excised just after each sample entered the resolving gel. A total of 144 proteins predicted by subcellular localization algorithms to be either located at the surface or secreted were identified in the overall fractions (Table 1). Of them, 33 were predicted to be lipoproteins (i.e., those proteins containing a type-II signal peptide), 83 were membrane proteins (i.e., those with one or more transmembrane domains), 19 were cell wall-anchored proteins (i.e., those possessing an LPXTG cell wall-anchoring motif) and nine were predicted to be type-I signal peptide secreted proteins (i.e., those released and lacking any known retention motif to the bacterial cell surface). Additionally, 680 proteins predicted to be of cytoplasmic origin were also identified (Table S1). The complete list of the proteins and peptides identified for each strain and replicate can be found in Supplementary File S1.

**Figure 1:**
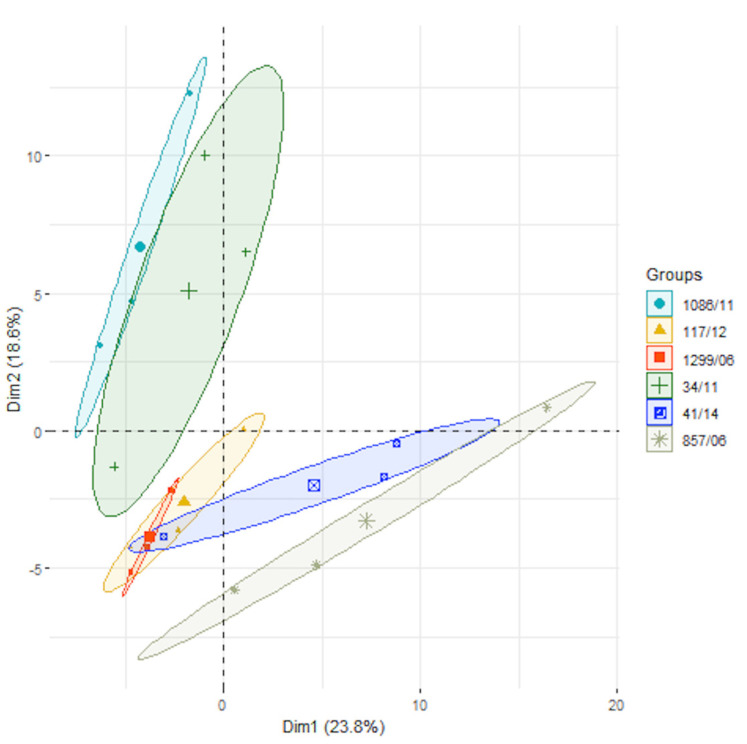
Principal component analysis (PCA) of the proteins identified in the six *Streptococcus suis* human clinical isolates.

Then, we carried out a label-free based analysis to compare the abundances of the predicted surface proteins in the six human isolates. A PCA analysis using the total proteins identified in the secretomes showed that principal component 1 (PC1) hardly discriminated among the six strains, with the second one (PC2) being capable of weakly discriminating isolates 34/11 and 1086/11 from the rest (Figure 1), thus confirming the visual differences obtained in the SDS-PAGE profiles.

**Figure 2:**
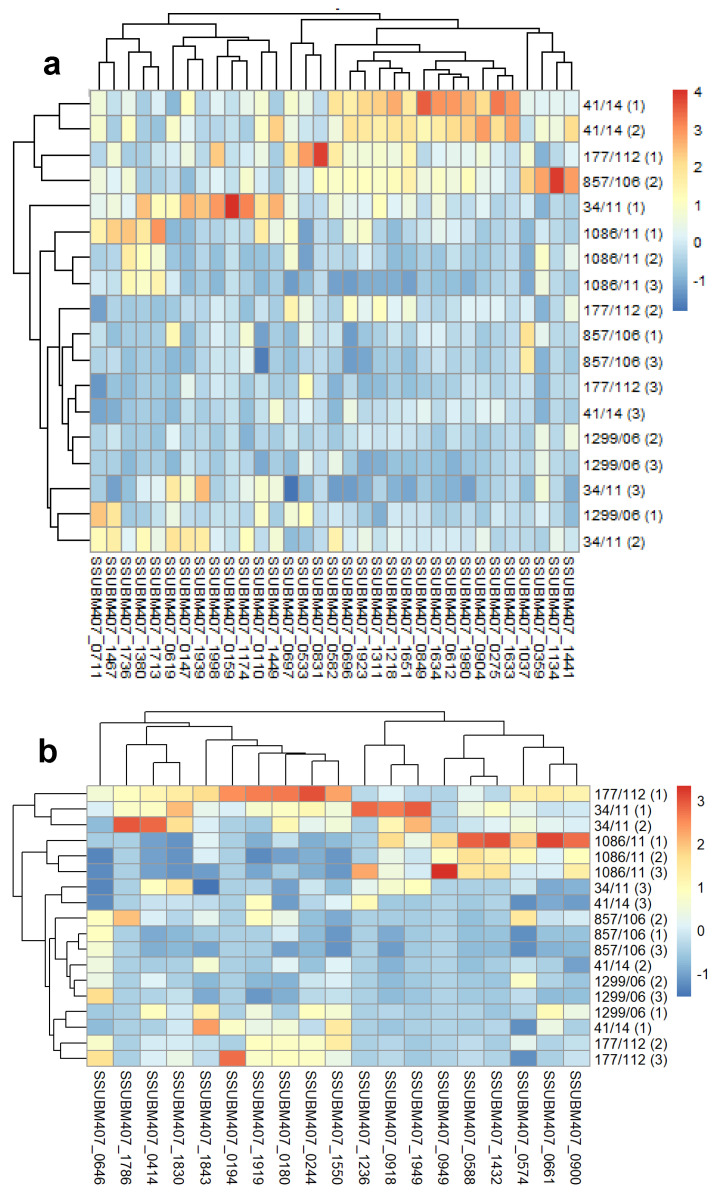

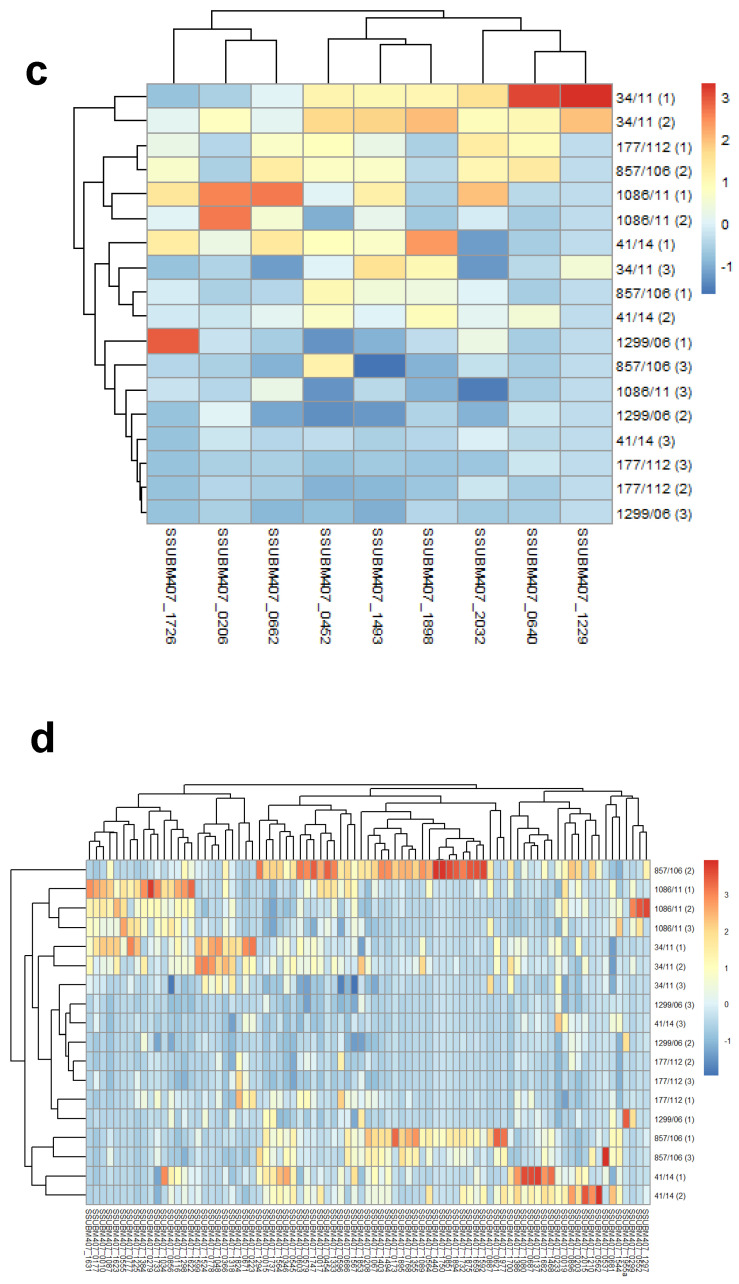
Hierarchically-clustered heatmaps of the z-scored predicted surface protein abundances in the six *Streptococcus suis* human clinical isolates: (**a**) lipoproteins, (**b**) cell wall proteins, (**c**) secreted proteins, (**d**) membrane proteins. The proteins are clustered in columns in each heatmap, and the isolates are shown in rows. The numbers in parenthesis in clinical isolates representing each of the three biological replicates.

After that, we calculated the normalized z-score abundances of the 144 surface predicted proteins grouped in the four aforementioned categories for an easier visualization (Figure 2; panel d is also available in the Supplementary Figures file in order to allow the easier reading of the labeling). Although the data were not very homogeneous because of the dispersion between the biological replicates (Figure S2), the hierarchically clustered heatmaps showed differences among the isolates with respect to different groups of proteins. Thus, the isolate 41/14 had significantly higher levels of lipoproteins than the rest; strains 34/11 and 1086/11 exhibited the highest levels of LPXTG cell wall proteins, sharing very high relative abundances of proteins SSUBM407_0574, SSUBM407_0588, SSUBM407_0918 and SSUBM407_1432; of note, the major pilin subunit SSUBM407_0414 was identified in all the isolates except in 1086/11; the abundances of the predicted signal peptide-I secreted proteins were higher in 34/11 than in the other five isolates, except for proteins SSUBM407_1726 and SSUBM407_0662; conversely, the distribution of the abundances of transmembrane proteins was less homogeneous, as expected from the high number of different proteins in this category. We searched for trends in up- or downregulated protein functions within each isolate, but we did not find any clear pattern. It seemed that in the strain 41/14, there was somehow an enrichment in amino acid ABC transporter proteins, or permease proteins in the isolate 857/06, but the results were not strong enough to conclude this.

Finally, we calculated the antigenicity of the proteins identified within the category of signal peptide-I secreted proteins (Table 2), but not those of the other predicted surface proteins, as they have been already studied in previous works [9,10,23,24,25]. Out of the nine identified proteins belonging to this category, seven were predicted to be clearly antigenic (VaxiJen score > 0.5), with four of them being found in the six isolates. Of note, the proteins SSUBM407_0452 and SSUBM407_0662 were identified from 116 and 169 peptide spectral matches (PSM), respectively, in all of the strains, thus indicating that they were the most abundant within this protein category. These results show them to be proteins with a potential for further immunogenicity studies.

This is the first work, to our knowledge, in which a proteomic comparison of th eexoproteomes of *S. suis* human clinical isolates was carried out. The analysis of secreted material is crucial in clinically relevant bacteria, as it may help elucidate possible roles in the infection or other biological processes [26,27,28,29]. We identified 144 proteins categorized as surface-attached or secreted extracellularly, as well as 680 cytoplasmic proteins. These included many proteins extensively reported to be externalized to the extracellular milieu or to anchor to the bacterial surface, including enolase, glyceraldehyde-3-phosphate dehydrogenase, fructose 1,6-bisphosphate aldolase and several ribosomal proteins. Many of these are known as “moonlighting proteins”, i.e., proteins that have dual functions lacking a canonical exporting or surface-retention motif, which nevertheless are located outside of the bacterial cytoplasm [8]. In a previous paper comparing the secreted fractions of *S. suis* strains isolated from animals, we showned that some of these proteins are immunoreactive [30]. Moreover, some of them have also been demonstrated to be immunogenic and to protect against infection in animal models. However, we cannot discard the supposition that these proteins are released to the exoproteome fractions after an uncontrolled cell lysis. We showed in previous works that *S. suis* grown at the mid-exponential phase undergoes <1% cell lysis [31]. Therefore, it is feasible that a residual proportion of the lysed cells could contaminate the extracellular fraction with cytoplasmic content.

It could be expected that, in the extracellular fraction, a higher proportion of signal peptide-I secreted proteins was found, as we identified 50% (9 out of 18) of those predicted in the BM407 genome. However, it is well known that not all of the proteins are always present in each experimental or biological condition. The presence of proteins with surface-anchoring motifs is not surprising in the exoproteome. Deliberately, we did not ultracentrifuge the culture media in order to separate the possible extracellular vesicles, as the purpose was to determine the protein content, either soluble or vesiculated, released by human isolates. Our research group reported the production of these ultrastructures in the pneumococcus, demonstrating that they contained abundant in lipoproteins and membrane proteins, but the presence of LPXTG cell wall proteins was rare [32]. However, cell wall proteins have been shown to be more extensively present in *S. suis* extracellular membrane vesicles [33]. This can explain why, in the present study, we found a high number of lipoproteins, membrane proteins and cell wall proteins, somewhat similar to that found in the “surfomes” previously described [10]. The proteins found in the exoproteomes were similar to those of the “surfomes” in these human clinical isolates, but they did differ in their abundances when comparing the two fractions, as revealed by the heatmap analysis. This could be explained if we assume that cell wall proteins could be released to the extracellular milieu in truncated forms after some proteolytic-mediated mechanisms. However, we did not find differences in the coverages between exoproteomes and “surfomes” (Supplementary File S2), except for the major pilin protein SSUBM407_0414. This protein was found in the exoproteomes of all of the strains, except in the 1086/11 isolate. In the “surfome” fractions, this protein was identified in the six isolates. We ignored the meaning of this finding.

## 4. Conclusions

In summary, this is the first report that described a comparative proteomic analysis of exoproteomes obtained from *S. suis* human clinical isolates. The work showed the presence of numerous proteins belonging to all of the possible categories of surface-attached or secreted proteins, many of which are also in common with other extracellular or surface fractions (“surfomes”, extracellular membrane vesicles). The bioinformatic analysis also revealed that most of the identified proteins predicted to be secreted in soluble form are antigenic. Some of these proteins could enter future pipelines for either vaccine or diagnostic studies.

## Figures and Tables

**Table 1:** Summary of the proteins in the exoproteomes of the six *Streptococcus suis* human clinical isolates, identified by GeLC-MS/MS analysis.

Protein Category ^a^	#^b^ Identified Proteins	#^b^ Predicted Proteins in *S. suis* BM407 Genome	Identified/Predicted (%)
Lipoprotein	33	40	82.5
Cell wall	19	20	95
Secreted	9	18	50
Membrane	83	479	17.3
Cytoplasmic	680	1375	49.4
Total	824	1932	42.7

^a^ The protein categories were defined according LocateP v2 predictions: lipoproteins were those predicted as lipid-anchored proteins with a type-II signal peptide; cell wall proteins were those with an LPXTG cell wall-anchoring motif; secreted proteins were predicted as those possessing a type-I signal peptide; membrane proteins were those with at least one predicted transmembrane domain; and cytoplasmic proteins predicted as those without any known exporting motif; ^b^ # indicates the number of either identified proteins in our proteomic analyses or those predicted in the *S. suis* BM407 genome.

**Table 2:** Predicted antigenicity of identified type-I signal peptide secreted proteins.

Protein Locus	Protein Annotation	VaxiJen Score ^a^	# Strains ^b^
SSUBM407_0206	Putative exported protein	0.6462	5
SSUBM407_0452	Putative exported protein	0.6648	6
SSUBM407_0640	Putative exported protein	0.4292	3
SSUBM407_0662	Putative N-acetylmuramoyl-L-alanine amidase	0.8672	6
SSUBM407_1229	Putative exported protein	0.5271	1
SSUBM407_1493	Putative exported protein	0.6369	6
SSUBM407_1726	LytR family regulatory protein	0.5444	3
SSUBM407_1898	UTP--glucose-1-phosphate uridylyltransferase	0.4444	6
SSUBM407_2032	Serine protease	0.5736	6

^a^ A protein was considered to be antigenic by VaxiJen if its score was equal to or higher than 0.5. ^b^ # indicates the number of *Streptococcus suis* human clinical isolates in which each of the proteins was identified by GeLC-MS/MS in the exoproteomes.

## Data Availability

The proteomics data presented in this study are available in Supplementary File S1.

## Supplementary Materials

The following are available online at https://www.mdpi.com/article/10.3390/microorganisms9061287/s1. Figure S1: SDS-PAGE of exoproteome fractions of the six *Streptococcus suis* human clinical isolates; Figure S2: Euclidean distances among the biological replicates of the proteins identified in the six *Streptococcus suis* human clinical isolates; Table S1: List and quantitative values of the identified surface proteins, as predicted by LocateP v2; Supplementary File S1: Complete list of the proteins and peptides identified in the six *Streptococcus suis* human clinical isolates; Supplementary File S2: Comparison of the sequence coverage of the predicted cell wall proteins between the exoproteomic and the “surfomic” analyses.
