# Supplementary material for: Comparative Exoproteome Analysis of Streptococcus suis Human Isolates
Source: Microorganisms. 2021 Jun 12;9(6):1287. doi: 10.3390/microorganisms9061287 (PMC8231589; doi:10.3390/microorganisms9061287)
Supplement: Supplementary file 1 [file microorganisms-09-01287-s001.zip › Supplementary Figures.pptx]

## Slide 1
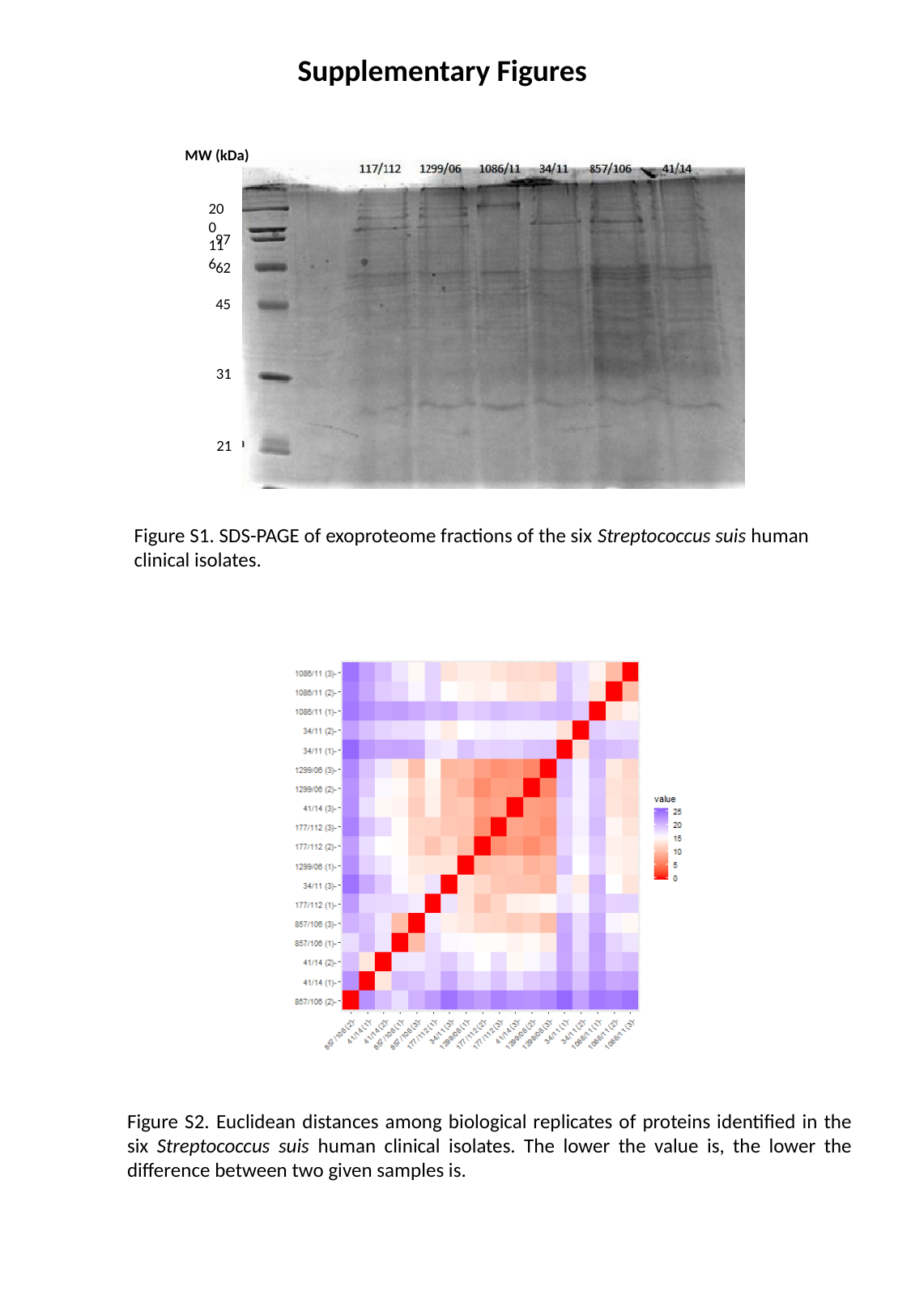

Supplementary Figures
MW (kDa)
200
116
97
 62
 45
31
 21
Figure S1. SDS-PAGE of exoproteome fractions of the six Streptococcus suis human clinical isolates.
Figure S2. Euclidean distances among biological replicates of proteins identified in the six Streptococcus suis human clinical isolates. The lower the value is, the lower the difference between two given samples is.

## Slide 2
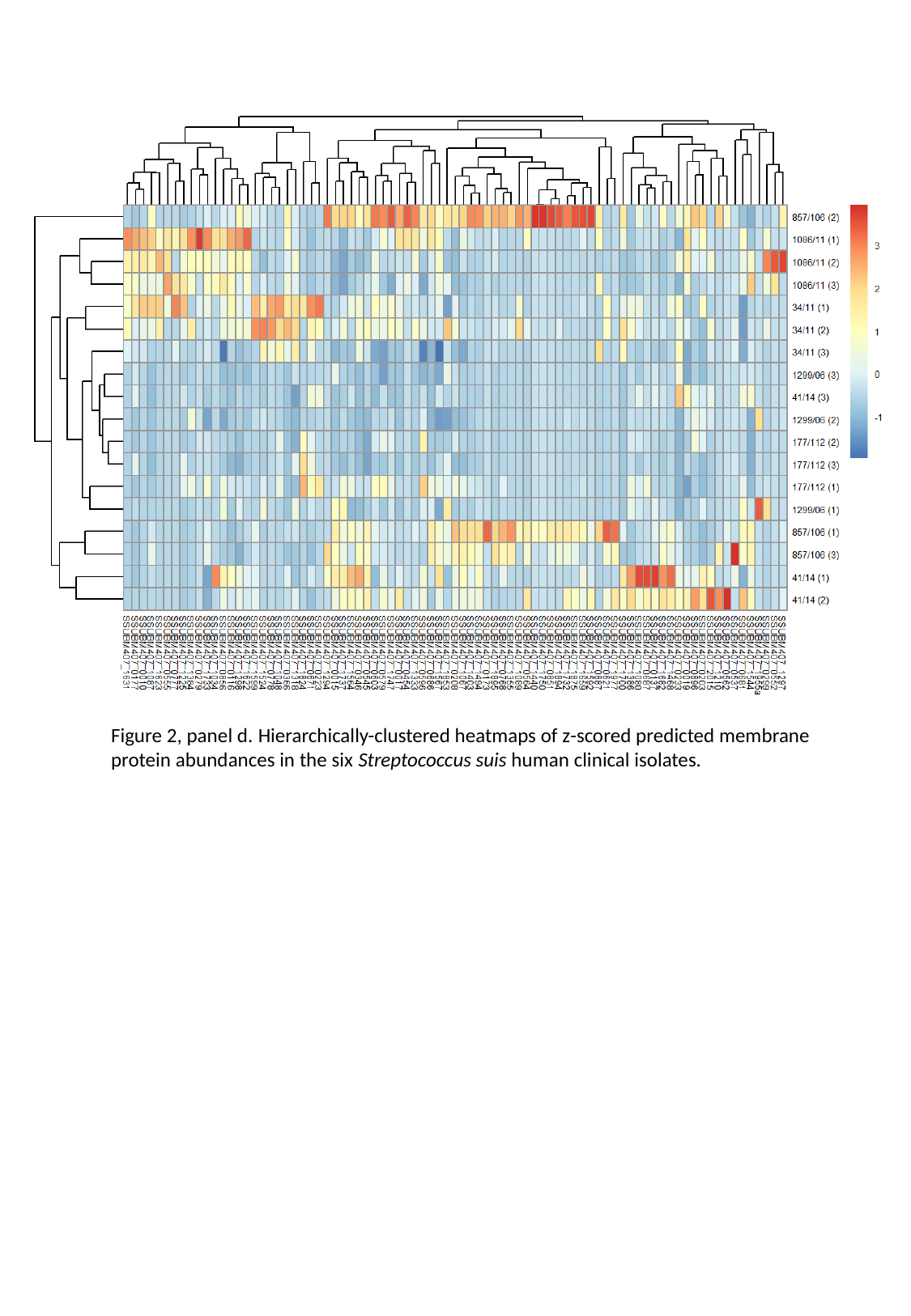

Figure 2, panel d. Hierarchically-clustered heatmaps of z-scored predicted membrane protein abundances in the six Streptococcus suis human clinical isolates.
