# Supplementary material for: Comparative Exoproteome Analysis of Streptococcus suis Human Isolates
Source: Microorganisms. 2021 Jun 12;9(6):1287. doi: 10.3390/microorganisms9061287 (PMC8231589; doi:10.3390/microorganisms9061287)
Supplement: Supplementary file 1 [file microorganisms-09-01287-s001.zip › Supplementary File 2.pdf]

Supplementary Dataset 2. Comparison of sequence coverage of predicted cell wall proteins that were identified in this study with that previously published for “surfomic” analysis (Prados de la Torre et al, Vaccines 2020, 8(2):188. doi: 10.3390/vaccines8020188). For such a comparison, peptides found in both proteomics datasets were mapped on the sequences of cell wall proteins. For each protein sequence, those highlighted in yellow represent the peptides found in the exoproteomes; those represented in green, the peptides found in the “surfomes”.

A0A0H3MXC9-Exoproteome

>tr|A0A0H3MXC9|A0A0H3MXC9_STRS4 Putative surface-anchored protein OS=Streptococcus suis (strain BM407) OX=568814 GN=SSUBM407_0180 PE=4 SV=1
METANKKFRYSIRKFKVGVGSVLIATCLLGAGVSTPTAFATTETSTATQVEAMAKVEEVQ
KLVKELEKELGELDKVPSYGDAQDYSYQKALWEEFLRIGKDNMDYASKMKADDKFFHKVK
GDLNDFKYQIKVENYIRQVAELRKKYPGDNTIEEEYNAHLKQDEGKSIASQEGATLRDYV
DREASEAMGRIKQRVAELEKSKQPQPSPADEPAPAPKEEDTPAPTPKVEDETQEPKTEEK
APETKEETPTPTPKEEGIPAPKEDEVPAPKEEETPAPKEEDTPAPDAAPAPAPTPEVDPA
PTPIPDTPKAEEEAPTPVPDTPAPKEDEVPAPIPDAPTPKVEEETQEPKTEEKAPETKEE
TPTPAPDAEPAPTPVPDTPAPDTPAPKEDEVPAPMPDAPAPKAEEEVPAPTPMPETPMDK
PKTDKVESDKQMPEAKQPEMEQPKAEDMPKEEMPKSEQPKAEDSAPKTAVPEVAPKTAEK
PKLDFTTKERKVEEALPIKEEIRYDASLPLGKSYLLQEGKAGKKVSVYQDVIVDGKVVAT
NLLSETVVEGQNRILVKGSLEMKKEEVKTTPSVQSNPTLSHKGAPSANKATLPATGEQRN
NLALVGLGLAGISLAVVATAINKKSKDQI

A0A0H3MXC9-Surfome

>tr|A0A0H3MXC9|A0A0H3MXC9_STRS4 Putative surface-anchored protein OS=Streptococcus suis (strain BM407) OX=568814 GN=SSUBM407_0180 PE=4 SV=1
METANKKFRYSIRKFKVGVGSVLIATCLLGAGVSTPTAFATTETSTATQVEAMAKVEEVQ
KLVKELEKELGELDKVPSYGDAQDYSYQKALWEEFLRIGKDNMDYASKMKADDKFFHKVK
GDLNDFKYQIKVENYIRQVAELRKKYPGDNTIEEEYNAHLKQDEGKSIASQEGATLRDYV
DREASEAMGRIKQRVAELEKSKQPQPSPADEPAPAPKEEDTPAPTPKVEDETQEPKTEEK
APETKEETPTPTPKEEGIPAPKEDEVPAPKEEETPAPKEEDTPAPDAAPAPAPTPEVDPA
PTPIPDTPKAEEEAPTPVPDTPAPKEDEVPAPIPDAPTPKVEEETQEPKTEEKAPETKEE
TPTPAPDAEPAPTPVPDTPAPDTPAPKEDEVPAPMPDAPAPKAEEEVPAPTPMPETPMDK
PKTDKVESDKQMPEAKQPEMEQPKAEDMPKEEMPKSEQPKAEDSAPKTAVPEVAPKTAEK
PKLDFTTKERKVEEALPIKEEIRYDASLPLGKSYLLQEGKAGKKVSVYQDVIVDGKVVAT
NLLSETVVEGQNRILVKGSLEMKKEEVKTTPSVQSNPTLSHKGAPSANKATLPATGEQRN
NLALVGLGLAGISLAVVATAINKKSKDQI

A0A0H3MTA4-Exoproteome

>tr|A0A0H3MTA4|A0A0H3MTA4_STRS4 Putative surface-anchored protein OS=Streptococcus suis (strain BM407) OX=568814 GN=SSUBM407_0194 PE=4 SV=1
MNKKIFLKSAIILLSVTCLINAIKALDSSGVSSSTGEIVNQDRIYNITLKVKTDGVYNDL
GGNVISENNVLSGISGTKINLKVSTNSGYRFVGYQSFLESGNSTDGLLPIENDSFEISDK
TGNVTVVAMFEKIPIDETIYFSDEFSTENINNYLLSENLIGKIAVSNGKLNIHAPGVSSI
KPILSRQIDESSTLGYEIQFSIQQIGEVKQWNTFRVVFKENSDGSVYALEFTGKAVSIKK
LSSIDAPNQTGEKYAETGHNLGSEEHRIRLVVRGDTVTVSDNEIPLLSYSSPENWEGATA
SIVFTPISNRSVSLDDIIIRQTRALRSLLVVSRIDGQEVTDIQPGSIRGNTSQVFVGDSL
PLEVIEKPGYQFIGFKDEFGNVVDLSTFSVPNDESDLVIYADFQTAEVVNRETKTFYIDS
IEGNDTNSGESETNAWKTLEQLRKNTLIAGDRVLLKRGSRFVGEDAALTFKGSGLEDAPI
LISSYGEGELPLLEAQGKIESVIKLYNQEYITIENLEITNLDPNFSTSFELNSNNNRSKI
LRGVHVIAEDYGVVHDIVLRNMYLHDINGNLNSKWNGGIFFDVYGTTVPTKYDGILIENN
YLERVDRSGIKLVGSTWANQNLKNNKNIPLNWYPSTNVVVRGNRIEKAGGDSITVRDTDG
ALIEYNISADARYQDTGYNAGIWPFQASNTVIQYNESFRTHGVQDGQGLDLDHVSNNSVM
QYNYSHDNEGGFMLIMNWYEQTSPTVRYNISQNDKDKIFELARGGAQGTAIYNNTIFSDS
KLTGRAGVIDMPSTSGGTGVKDIFLFNNIFYFTDGEKMFVEASDAGKYKDSIHFYNNAYV
GVEVPDDPKAITEGITLKGVGTGPTENKSMIANAGKFLTGQLDGYRLPENSSLAKLGVSK
EEAISYFYKKLGVQPTIDFQDNGSLTMSPTEAFALARQTNSVDAIARVYPAIEGVTYDTD
FFGETLSSENLSVGAAQEKKSVFEEKISDIEISDVKTGIGMRYKRSNQLADVNLSIRLIN
KDELQYTINANNPTFSYEIQLQKNFGEYMDLSESVTITIPIEADKKILNVLKINKNEQTE
SVKELPYRIENNKIILETDELGSFIIEYQMEKIKKENNTNATISESGKSSLGLQNKENSD
VQAVGDKDYFSGLELSNKGEELSREDSKLMGNSINNLQTIKESESIFEGNLGRNKELSDM
YQKSLPKTGESNQAYIVLIGLFSLFVVLICQIFKKSID

A0A0H3MTA4-Surfome

>tr|A0A0H3MTA4|A0A0H3MTA4_STRS4 Putative surface-anchored protein OS=Streptococcus suis (strain BM407) OX=568814 GN=SSUBM407_0194 PE=4 SV=1
MNKKIFLKSAIILLSVTCLINAIKALDSSGVSSSTGEIVNQDRIYNITLKVKTDGVYNDL
GGNVISENNVLSGISGTKINLKVSTNSGYRFVGYQSFLESGNSTDGLLPIENDSFEISDK
TGNVTVVAMFEKIPIDETIYFSDEFSTENINNYLLSENLIGKIAVSNGKLNIHAPGVSSI
KPILSRQIDESSTLGYEIQFSIQQIGEVKQWNTFRVVFKENSDGSVYALEFTGKAVSIKK
LSSIDAPNQTGEKYAETGHNLGSEEHRIRLVVRGDTVTVSDNEIPLLSYSSPENWEGATA
SIVFTPISNRSVSLDDIIIRQTRALRSLLVVSRIDGQEVTDIQPGSIRGNTSQVFVGDSL
PLEVIEKPGYQFIGFKDEFGNVVDLSTFSVPNDESDLVIYADFQTAEVVNRETKTFYIDS
IEGNDTNSGESETNAWKTLEQLRKNTLIAGDRVLLKRGSRFVGEDAALTFKGSGLEDAPI
LISSYGEGELPLLEAQGKIESVIKLYNQEYITIENLEITNLDPNFSTSFELNSNNNRSKI
LRGVHVIAEDYGVVHDIVLRNMYLHDINGNLNSKWNGGIFFDVYGTTVPTKYDGILIENN
YLERVDRSGIKLVGSTWANQNLKNNKNIPLNWYPSTNVVVRGNRIEKAGGDSITVRDTDG
ALIEYNISADARYQDTGYNAGIWPFQASNTVIQYNESFRTHGVQDGQGLDLDHVSNNSVM
QYNYSHDNEGGFMLIMNWYEQTSPTVRYNISQNDKDKIFELARGGAQGTAIYNNTIFSDS
KLTGRAGVIDMPSTSGGTGVKDIFLFNNIFYFTDGEKMFVEASDAGKYKDSIHFYNNAYV
GVEVPDDPKAITEGITLKGVGTGPTENKSMIANAGKFLTGQLDGYRLPENSSLAKLGVSK
EEAISYFYKKLGVQPTIDFQDNGSLTMSPTEAFALARQTNSVDAIARVYPAIEGVTYDTD
FFGETLSSENLSVGAAQEKKSVFEEKISDIEISDVKTGIGMRYKRSNQLADVNLSIRLIN
KDELQYTINANNPTFSYEIQLQKNFGEYMDLSESVTITIPIEADKKILNVLKINKNEQTE
SVKELPYRIENNKIILETDELGSFIIEYQMEKIKKENNTNATISESGKSSLGLQNKENSD
VQAVGDKDYFSGLELSNKGEELSREDSKLMGNSINNLQTIKESESIFEGNLGRNKELSDM
YQKSLPKTGESNQAYIVLIGLFSLFVVLICQIFKKSID

A0A0H3MTC5-Exoproteome

>tr|A0A0H3MTC5|A0A0H3MTC5_STRS4 Putative surface-anchored protein OS=Streptococcus suis (strain BM407) OX=568814 GN=SSUBM407_0244 PE=4 SV=1
MSKQKVVSSLLLSTVVLGGLAFYSTTTVKAESLEPDVTSVNASDATNKPVVPNEEGGDFLAEEELEDDDT
LEEELNEKAEEVTEPSSPEALLQPRAMMSDSETSGMEEIPMNDEPSDNTEEKVEKQQSPLIQTSNADYKS
GKDQEKLRTSVSINLLKAEEGQIQWKVTFDTSEWSFNVKHGGVYFILPNGLDLTKIVDNNQHDITASFPT
DINDYRNSGQEKYRFFSSKQGLDNENGFNSQWNWSAGQANPSETVNSWKSGNRLSKIYFINQITDTTELT
YTLTAKVTEPNQQSFPLLAVMKSFTYTNSKSTEVTSLGAREITLEKEKTLPPKENPKPEPEAPKPDAPQA
PSAPESPTEEPKKEDAPQTPQAPSTPEKQPEVPESPNPETPDAPSTPKDEPQAPSIPEEKPQVPEEPKQE
APSAPSTPEKQPEAPESPTEEPKKEDAPAPSTPEKQPEVPESPNPETPDAPSTPKDEPQVPSIPEEQPKE
TPAPEEPKKEDTPQTPQAPSTPKEEAPKEEVPTPPAPSVPEEQPKETPTPEVPKQEDVQPEAPKSDKVES
DKQMPETKKPDMKQPKADDMPKEQKPKADEPKAEQPQMDKPQMEAPKKDSEAPKSDKVETDKQLPETKQP
DMKQPKADDMPKEQKPKADEPKAEQPQMDKPQMEAPKKDSEAPKSDKVETDKPMPETKQPDMKQPKADKP
EAEKAQMPRTEGMKPESKASMMPKAEAPKATLPNTGEASSAIGWLGGALATLATGLYLFKNKKEE

A0A0H3MTC5-Surfome

>tr|A0A0H3MTC5|A0A0H3MTC5_STRS4 Putative surface-anchored protein OS=Streptococcus suis (strain BM407) OX=568814 GN=SSUBM407_0244 PE=4 SV=1
MSKQKVVSSLLLSTVVLGGLAFYSTTTVKAESLEPDVTSVNASDATNKPVVPNEEGGDFLAEEELEDDDT
LEEELNEKAEEVTEPSSPEALLQPRAMMSDSETSGMEEIPMNDEPSDNTEEKVEKQQSPLIQTSNADYKS
GKDQEKLRTSVSINLLKAEEGQIQWKVTFDTSEWSFNVKHGGVYFILPNGLDLTKIVDNNQHDITASFPT
DINDYRNSGQEKYRFFSSKQGLDNENGFNSQWNWSAGQANPSETVNSWKSGNRLSKIYFINQITDTTELT
YTLTAKVTEPNQQSFPLLAVMKSFTYTNSKSTEVTSLGAREITLEKEKTLPPKENPKPEPEAPKPDAPQA
PSAPESPTEEPKKEDAPQTPQAPSTPEKQPEVPESPNPETPDAPSTPKDEPQAPSIPEEKPQVPEEPKQE
APSAPSTPEKQPEAPESPTEEPKKEDAPAPSTPEKQPEVPESPNPETPDAPSTPKDEPQVPSIPEEQPKE
TPAPEEPKKEDTPQTPQAPSTPKEEAPKEEVPTPPAPSVPEEQPKETPTPEVPKQEDVQPEAPKSDKVES
DKQMPETKKPDMKQPKADDMPKEQKPKADEPKAEQPQMDKPQMEAPKKDSEAPKSDKVETDKQLPETKQP
DMKQPKADDMPKEQKPKADEPKAEQPQMDKPQMEAPKKDSEAPKSDKVETDKPMPETKQPDMKQPKADKP
EAEKAQMPRTEGMKPESKASMMPKAEAPKATLPNTGEASSAIGWLGGALATLATGLYLFKNKKEE

A0A0H3MTL3-Exoproteome

>tr|A0A0H3MTL3|A0A0H3MTL3_STRS4 Major pilus subunit OS=Streptococcus suis (strain BM407) OX=568814 GN=SSUBM407_0414 PE=4 SV=1
MKKLQQFFTSLVAVLTVFAFGSTALAQTVDSGKSGAGTITVSNASQGQTYTAYKLFDATVTTDGSGISYK
LPAGKTATNFGGDTWFEVDSKGNITAKEGATVSSEAFAAWAKNFGTEVTSATANDNSLVFTHLTFGYYFV
TSSLGAVLTVDSTTPNATVIDKNTTNPTIPDSNNGGGKKILSNGATTSETTAKIGDTINFQIKFNATNYV
TKDRQTKQIVSYTIEDSPTALAIDQNSVNVKVDGVDITAKISKTFDATGKMNLVITWADAAASNKTIYNS
PAEVIITYSAVVTKDAKEGEATNSATIGYNTIDNPTTPPTPVDPDKPTETTKVTTHRFTLKKTNNVGETL
TGAEFKLYDAANNGTEIKVVKESDGVYRVAQADEQGVVIEAGEVVIKGLKHSTTYYLEEMKAPNGYNILT
ERQSIEVKENNTAQANIVNKKGGVLPSTGAIGTTLFYLVGSILLLVALVYTISKRRMNNI

A0A0H3MTL3-Surfome

>tr|A0A0H3MTL3|A0A0H3MTL3_STRS4 Major pilus subunit OS=Streptococcus suis (strain BM407) OX=568814 GN=SSUBM407_0414 PE=4 SV=1
MKKLQQFFTSLVAVLTVFAFGSTALAQTVDSGKSGAGTITVSNASQGQTYTAYKLFDATVTTDGSGISYK
LPAGKTATNFGGDTWFEVDSKGNITAKEGATVSSEAFAAWAKNFGTEVTSATANDNSLVFTHLTFGYYFV
TSSLGAVLTVDSTTPNATVIDKNTTNPTIPDSNNGGGKKILSNGATTSETTAKIGDTINFQIKFNATNYV
TKDRQTKQIVSYTIEDSPTALAIDQNSVNVKVDGVDITAKISKTFDATGKMNLVITWADAAASNKTIYNS
PAEVIITYSAVVTKDAKEGEATNSATIGYNTIDNPTTPPTPVDPDKPTETTKVTTHRFTLKKTNNVGETL
TGAEFKLYDAANNGTEIKVVKESDGVYRVAQADEQGVVIEAGEVVIKGLKHSTTYYLEEMKAPNGYNILT
ERQSIEVKENNTAQANIVNKKGGVLPSTGAIGTTLFYLVGSILLLVALVYTISKRRMNNI

A0A0H3N2T0-Exoproteome

>tr|A0A0H3N2T0|A0A0H3N2T0_STRS4 Membrane dipeptidase OS=Streptococcus suis (strain BM407) OX=568814 GN=SSUBM407_0574 PE=3 SV=1
MKKTFVRIATFLMLVCLFPVQLVQACSGFIIGKGLTTDGSILYGRTEDYPYPPNNGAHNKNYIVVPATAY
AKGDMLVDESFGFTAPHLANEFKYTSTPDAARGDGSNGNFGAHGFNEKGVSMTATVTAIPNKKVLAVDPL
VTAGGLGEAILIDYVLPRVTSAREGIELIAKTIDEKGSAEGNIIVIADKNEVWYMEILSGHQYVAIKFPE
DKYAIFANTYYLGHVDFTDTENVIASAKVEEVAKQAENYMMVDGKFHIAKSYGPENYADGDRSRTYAGIK
LLDPASSVTYEDAVYDLLRQPTDPSRRFSLQDTFALQRNRFEHLPEFRPDDEAGKVKQGDNGANDQAADA
TYKYALGNENVIDAHVYQINSSLPSAFGGTVWLGLAQTRNTPYVPFYGIVTDTYEAFKNRSASYDTNSWY
WTVQNIDKMAISHPELFGTSIQEKWIALEKEWIASQAALDAQYAGLSEDAAVALAPTVTEATLARSAEIF
AQLKAVEAEMMAKIEAATTPSSSSTEPSTSTEPSSSGTETSTSTSQSTSDSNTGGATDTSSSSRTVVPSD
KKVTPTNKKGKSSLPSTGEQVSLLLVALGVAGILTAIFLHRKKSSKE

A0A0H3N2T0-Surfome

>tr|A0A0H3N2T0|A0A0H3N2T0_STRS4 Membrane dipeptidase OS=Streptococcus suis (strain BM407) OX=568814 GN=SSUBM407_0574 PE=3 SV=1
MKKTFVRIATFLMLVCLFPVQLVQACSGFIIGKGLTTDGSILYGRTEDYPYPPNNGAHNKNYIVVPATAY
AKGDMLVDESFGFTAPHLANEFKYTSTPDAARGDGSNGNFGAHGFNEKGVSMTATVTAIPNKKVLAVDPL
VTAGGLGEAILIDYVLPRVTSAREGIELIAKTIDEKGSAEGNIIVIADKNEVWYMEILSGHQYVAIKFPE
DKYAIFANTYYLGHVDFTDTENVIASAKVEEVAKQAENYMMVDGKFHIAKSYGPENYADGDRSRTYAGIK
LLDPASSVTYEDAVYDLLRQPTDPSRRFSLQDTFALQRNRFEHLPEFRPDDEAGKVKQGDNGANDQAADA
TYKYALGNENVIDAHVYQINSSLPSAFGGTVWLGLAQTRNTPYVPFYGIVTDTYEAFKNRSASYDTNSWY
WTVQNIDKMAISHPELFGTSIQEKWIALEKEWIASQAALDAQYAGLSEDAAVALAPTVTEATLARSAEIF
AQLKAVEAEMMAKIEAATTPSSSSTEPSTSTEPSSSGTETSTSTSQSTSDSNTGGATDTSSSSRTVVPSD
KKVTPTNKKGKSSLPSTGEQVSLLLVALGVAGILTAIFLHRKKSSKE

A0A0H3MU32-Exoproteome

>tr|A0A0H3MU32|A0A0H3MU32_STRS4 Putative surface-anchored protein OS=Streptococcus suis (strain BM407) OX=568814 GN=sao PE=4 SV=1
MNTKKWRTSLLIPGIVLFGTVALVNNVSAQEVKNTIISAKQPDGGQATSKAVNVKIPAVVRLFGRELLEN
EFKFELREANGEELPVLDTAQNTKEGQVRFKNLSFDKPGKYWYTISEVKDELGGIEYDSKYIVAKITVED
RNGQLQAMIEFIDNDNVFNNFYTPAPAAASLSIKKVLEGRTLNTGEFEFVLKNEKGDEIEKVSNQADGSV
NFSALTFTKEGTYTYTVSEVDGGLGDIIYDKSDIKATVTVKDNNHGQLVSTVTYENSDQIFENILNPGKL
IAPTTDSVITDNEVSKEAMTGKEKGNIEPPKEQIANEEKDNIEASEKQMPSIVNDMVVTPEKQMTNKEND
KVVISEKQMPSVVNENAVTPEKQMTNKENDNIETSEKQMPSVVNENAVTPEKQMTNKEKDNIETSEKQMP
SIVNDMVVTPQEQMANKENDKVVISEKQMPSIVNDMVVTPQEQMANKENDKVVISEKQMPSIVNDMVVTP
QEQMANKENDKVVISEKQMPSIVNDMVVTPQEQMANKENDKVETSEKQMPVNEKDNAVTPEKQMANKEKE
NIETSKKQIPVNENNQNGTVEENSNTKPTTEKTDKQETSTFKTETAKQILPVTGEKGSLWLLTSGIIGLA
IALFTRKRK

A0A0H3MU32-Surfome

>tr|A0A0H3MU32|A0A0H3MU32_STRS4 Putative surface-anchored protein OS=Streptococcus suis (strain BM407) OX=568814 GN=sao PE=4 SV=1
MNTKKWRTSLLIPGIVLFGTVALVNNVSAQEVKNTIISAKQPDGGQATSKAVNVKIPAVVRLFGRELLEN
EFKFELREANGEELPVLDTAQNTKEGQVRFKNLSFDKPGKYWYTISEVKDELGGIEYDSKYIVAKITVED
RNGQLQAMIEFIDNDNVFNNFYTPAPAAASLSIKKVLEGRTLNTGEFEFVLKNEKGDEIEKVSNQADGSV
NFSALTFTKEGTYTYTVSEVDGGLGDIIYDKSDIKATVTVKDNNHGQLVSTVTYENSDQIFENILNPGKL
IAPTTDSVITDNEVSKEAMTGKEKGNIEPPKEQIANEEKDNIEASEKQMPSIVNDMVVTPEKQMTNKEND
KVVISEKQMPSVVNENAVTPEKQMTNKENDNIETSEKQMPSVVNENAVTPEKQMTNKEKDNIETSEKQMP
SIVNDMVVTPQEQMANKENDKVVISEKQMPSIVNDMVVTPQEQMANKENDKVVISEKQMPSIVNDMVVTP
QEQMANKENDKVVISEKQMPSIVNDMVVTPQEQMANKENDKVETSEKQMPVNEKDNAVTPEKQMANKEKE
NIETSKKQIPVNENNQNGTVEENSNTKPTTEKTDKQETSTFKTETAKQILPVTGEKGSLWLLTSGIIGLA
IALFTRKRK

A0A0H3MU94-Exoproteome

>tr|A0A0H3MU94|A0A0H3MU94_STRS4 Putative surface-anchored zinc carboxypeptidase OS=Streptococcus suis (strain BM407) OX=568814 GN=SSUBM407_0646 PE=4 SV=1
MKLSYKKRLLNQVLLASTVLLAASLAQGTVFANTEEIPPTTNETVTPLPEETPITKTSTSEATDNLVEGK
ETEKQTEEIADTSPASVSTEEETTSSEPNAEETTLRTANNDNQNTTEEKPAVPTIDTITLETKTVYLSEA
VTITESVTLPDTTEKIEWILDGKPISEWKTWNLKEGDFTGDTFITVEESRQDNQLHLNIQLAALFGEDLS
KRTPSNIRRTYRHFIKDMLLEGTSADGNLIISKTLHFRPYEAYRTHEEMLTEIEETKNNAATDRLVRIES
IGQSAEGRDIKMAVVAKNQASIDKYLTETTPLMLTQPDQMLKQLQAGTFDYRLPILINNTHADEQPGIDV
VTSLFKEFAQKDTITFPSTDADGNPVTLHLKVTDLLDKFIFLFNFTENPDGDVKNLRSLVNGLDPNRDTG
FQVNPETQAIVRQIHKWNPISVLDIHGFVSGFLIEPATPPHDPNFEYDLLADIMLEKAHEMGRAGIANSK
YERYTIPKVHWGDGWDDSFSGYTAVYAMYHGILGHTIEIPEGNQESFKAGFFAVLGGVHNMATKPDSLME
MRLKYYSRGVNKVEDPKAESELVGPDGAVVGRVKKDQPKFFPDYYVIPMTLDKHNDMQEAFKMIEYFNRN
GVVVKELTEDVGNFRKGDLVVDMAQAKRGFANHVLYAGSDESAWGAMYAELVVNFPDMKGFSAKAVFEEN
TFSGKLGSITWTKAPRTTEIDFKAPYYVVANTSESAVQAINQAIKSGAKVYLTDDGYIMETNQFSHLLDT
YALYGEPLYKKPLGQELKAMKVYAPSHSYSWAGDFAILANAALAVERMGFEIVNSADEADAIILESDQFD
ASVFGKKPIIIVGGVAMQKLEELGILAGFNAEQFTDGGDYEGLMQAIIDDKDPLTSGYAMNGLFYSNSGN
WIEGIPEGFKTLVKIADKDYYIAGWWPGHDKLANKIVAIAGNYQDQPVFIYAGNPTNKVHPVHFFRWVSN
ALFGSQLASLEDLPAVEILVPQPEMPKNILDEAPKTTTVVHTTKSTEAKQLPQTGEKTNYIAIALGSLLL
GSIALRRKERS

A0A0H3MU94-Surfome

>tr|A0A0H3MU94|A0A0H3MU94_STRS4 Putative surface-anchored zinc carboxypeptidase OS=Streptococcus suis (strain BM407) OX=568814 GN=SSUBM407_0646 PE=4 SV=1
MKLSYKKRLLNQVLLASTVLLAASLAQGTVFANTEEIPPTTNETVTPLPEETPITKTSTSEATDNLVEGK
ETEKQTEEIADTSPASVSTEEETTSSEPNAEETTLRTANNDNQNTTEEKPAVPTIDTITLETKTVYLSEA
VTITESVTLPDTTEKIEWILDGKPISEWKTWNLKEGDFTGDTFITVEESRQDNQLHLNIQLAALFGEDLS
KRTPSNIRRTYRHFIKDMLLEGTSADGNLIISKTLHFRPYEAYRTHEEMLTEIEETKNNAATDRLVRIES
IGQSAEGRDIKMAVVAKNQASIDKYLTETTPLMLTQPDQMLKQLQAGTFDYRLPILINNTHADEQPGIDV
VTSLFKEFAQKDTITFPSTDADGNPVTLHLKVTDLLDKFIFLFNFTENPDGDVKNLRSLVNGLDPNRDTG
FQVNPETQAIVRQIHKWNPISVLDIHGFVSGFLIEPATPPHDPNFEYDLLADIMLEKAHEMGRAGIANSK
YERYTIPKVHWGDGWDDSFSGYTAVYAMYHGILGHTIEIPEGNQESFKAGFFAVLGGVHNMATKPDSLME
MRLKYYSRGVNKVEDPKAESELVGPDGAVVGRVKKDQPKFFPDYYVIPMTLDKHNDMQEAFKMIEYFNRN
GVVVKELTEDVGNFRKGDLVVDMAQAKRGFANHVLYAGSDESAWGAMYAELVVNFPDMKGFSAKAVFEEN
TFSGKLGSITWTKAPRTTEIDFKAPYYVVANTSESAVQAINQAIKSGAKVYLTDDGYIMETNQFSHLLDT
YALYGEPLYKKPLGQELKAMKVYAPSHSYSWAGDFAILANAALAVERMGFEIVNSADEADAIILESDQFD
ASVFGKKPIIIVGGVAMQKLEELGILAGFNAEQFTDGGDYEGLMQAIIDDKDPLTSGYAMNGLFYSNSGN
WIEGIPEGFKTLVKIADKDYYIAGWWPGHDKLANKIVAIAGNYQDQPVFIYAGNPTNKVHPVHFFRWVSN
ALFGSQLASLEDLPAVEILVPQPEMPKNILDEAPKTTTVVHTTKSTEAKQLPQTGEKTNYIAIALGSLLL
GSIALRRKERS

A0A0H3MUB3-Exoproteome

>tr|A0A0H3MUB3|A0A0H3MUB3_STRS4 Putative surface-anchored protein OS=Streptococcus suis (strain BM407) OX=568814 GN=SSUBM407_0661 PE=4 SV=1
MKKKAIVKPLLMSGIFLALLGGATLPSGAVPVVAAETSQSTTYHLTDDEKVAVREYIQAKMTIDMQEYRL
AFLEGMMEEMASGSAEAAWDEEIADLKANLTAEQVVVLDELEANLIGSIAQHYHYLFETLTVAGKSGREE
AAAIVSKYESEDDASTPEAELAALKYAREVIVELLNKESAAIDNYIAYAEATGQELAGLLESGNSNLESI
TSATIGYGQALATASQPKFPYDFSEMDRQIAELTASLQSKVEDKSTAKTENTGVQTSQSATNGSNDLQTV
PDQGGGQISDVATGKGNISEAGQKKVIPNDNAKVLPKTSGKSSLPLTVLGLITIFAGWLLTNKQEEK

A0A0H3MUB3-Surfome

>tr|A0A0H3MUB3|A0A0H3MUB3_STRS4 Putative surface-anchored protein OS=Streptococcus suis (strain BM407) OX=568814 GN=SSUBM407_0661 PE=4 SV=1
MKKKAIVKPLLMSGIFLALLGGATLPSGAVPVVAAETSQSTTYHLTDDEKVAVREYIQAKMTIDMQEYRL
AFLEGMMEEMASGSAEAAWDEEIADLKANLTAEQVVVLDELEANLIGSIAQHYHYLFETLTVAGKSGREE
AAAIVSKYESEDDASTPEAELAALKYAREVIVELLNKESAAIDNYIAYAEATGQELAGLLESGNSNLESI
TSATIGYGQALATASQPKFPYDFSEMDRQIAELTASLQSKVEDKSTAKTENTGVQTSQSATNGSNDLQTV
PDQGGGQISDVATGKGNISEAGQKKVIPNDNAKVLPKTSGKSSLPLTVLGLITIFAGWLLTNKQEEK

A0A0H3MV17-Exoproteome

>tr|A0A0H3MV17|A0A0H3MV17_STRS4 Putative 5'-nucleotidase OS=Streptococcus suis (strain BM407) OX=568814 GN=sntC PE=3 SV=1
MPKKGLFMKKKKILLPVMSTLLLAPFVLAQQVQAAETTTAATTTNQPATTDATATVPATTDATATVPATS
VENVATEETVVPAAEETVEAVIIHTNDVHGRILEEKNVIGDAKAAAVIEEERAKVENTIVVDAGDAFQGL
PISNSTKGEDRANIMNQVGYDAMAVGNHEFDFGMDQAIKYKETLNFPLLSANTYVNGARVFEASTIVDKT
PTVVGDEFVVIGVTTPETATKTHPKNVEGVTFTDPVTEVNKVIDEVEARALADNRVYKNYIILAHLGVDS
TTPVEWRGSTLAEALSKNSKLAGKRVIVIDGHSHTVEATTYGDNVTYNQTGSYLNNIGKVTLKSDKLLGE
ASLISAADTKNVTPNAKIAALVDEIKAKYEAENAQVVIENNPVELNGDRSNVRVRETNLGNAVTDAIYAY
GQTGFSNKTSLAVTNGGGLRATIAKDQPVTKGDIIAVLPFGNIVSQITVTGQQIYDMFTKSLSSTLQVNP
ETGEMLLDENGMPLFEASGGFLHISGANVFYDPTLPVEERVLLIGILNPETGEYDALDLEKTYYLATNDF
LAAGGDGYTMLGGAREEGPSMDSVFAEYLKTADLSAYEVVNPYSRIIPVNSSIDTDEDGYPDFIEILLDT
DPENPASNPETVPAENTDSPSNQVQNTSATDKKAPVDSPKVGDKKTEVASPAKTTKAGVLPNTGDQMNLT
LSLFGLGLAGLAVAVGRRKEN

A0A0H3MV17-Surfome

>tr|A0A0H3MV17|A0A0H3MV17_STRS4 Putative 5'-nucleotidase OS=Streptococcus suis (strain BM407) OX=568814 GN=sntC PE=3 SV=1
MPKKGLFMKKKKILLPVMSTLLLAPFVLAQQVQAAETTTAATTTNQPATTDATATVPATTDATATVPATS
VENVATEETVVPAAEETVEAVIIHTNDVHGRILEEKNVIGDAKAAAVIEEERAKVENTIVVDAGDAFQGL
PISNSTKGEDRANIMNQVGYDAMAVGNHEFDFGMDQAIKYKETLNFPLLSANTYVNGARVFEASTIVDKT
PTVVGDEFVVIGVTTPETATKTHPKNVEGVTFTDPVTEVNKVIDEVEARALADNRVYKNYIILAHLGVDS
TTPVEWRGSTLAEALSKNSKLAGKRVIVIDGHSHTVEATTYGDNVTYNQTGSYLNNIGKVTLKSDKLLGE
ASLISAADTKNVTPNAKIAALVDEIKAKYEAENAQVVIENNPVELNGDRSNVRVRETNLGNAVTDAIYAY
GQTGFSNKTSLAVTNGGGLRATIAKDQPVTKGDIIAVLPFGNIVSQITVTGQQIYDMFTKSLSSTLQVNP
ETGEMLLDENGMPLFEASGGFLHISGANVFYDPTLPVEERVLLIGILNPETGEYDALDLEKTYYLATNDF
LAAGGDGYTMLGGAREEGPSMDSVFAEYLKTADLSAYEVVNPYSRIIPVNSSIDTDEDGYPDFIEILLDT
DPENPASNPETVPAENTDSPSNQVQNTSATDKKAPVDSPKVGDKKTEVASPAKTTKAGVLPNTGDQMNLT
LSLFGLGLAGLAVAVGRRKEN

A0A0H3MV42-Exoproteome

>tr|A0A0H3MV42|A0A0H3MV42_STRS4 Putative glucan-binding surface-anchored protein OS=Streptococcus suis (strain BM407) OX=568814 GN=SSUBM407_0949 PE=4 SV=1
MTKTCNHHFLVNQEKGEKHVFRKSKKYRTLCSVALGTMVTAVVAWGGTVAHADEVVLSVDSTIQRTENPA
TNLPEAQPNPVSEQTESLGSTGKSNGAIAVTVPHETVTQTVEQAKAEGVSTVEDSPMDLGNTTSAAETNQ
QISKAEADAQNQVVAINEVTETYKADKAAYVDEKARIEQENKELSQAYEGANQTGKETNAWVDSKVNDLK
SQYADADVTVKEQVVSSGKGTSVLDYTDYGKAVETIQSTNEQAVADYLTKKTKADDIVAKNQAIQKENEA
GLAKAKADNEAIERRNQAGQAAVDAENRAGQAAVDQANQEKQQLVSDRAVEIEVITKRNQAKEVAARKEN
EAIDAYNAKEMERYQRDLAEISKGEEGYISEALAQALNLNNGEPQAQHGAITRNPNQIISTGDAMLGGYS
RILDSTGFFVYDSFKTGETLSFNYQNLQHARFDGKKISRVTYDITNLVSPAGTDAVKLVVPNDPTEGFIA
YRNDGNGDWRTDKMEFRVVAKYFLEDGSQVTFSKEKPGVFTHSSLNHNDIGLEYVKDSSGKFVPINGSTV
QVTNEGLARSLGSNRASDLNLPEEWDTTSSRYAYKGAIVSTVTSGNTYTVTFGQGDMPQNVGLSYWFALN
TLPVARTVTPYSPKPHVTVELEPVPEPITVTPDVYTPKTFTPEKPVTFTPKPLEEVVQPSLTLTKVTLPV
KPIPKELPTPPQVPTIHYHAYRLTTTPEIMKEVINSDQANLHDKTVAKDSTVIYPLTVDALSPKRTQTTS
LIFEDYLPAGYLFDKETTQKENGNYALSFDESKNFVTLTAKETLLQEVNQDLTKSYQLTAPKLYGTVQND
GATYSNSYKLLVNKGTANSYTVTSNIVTVRTPGDGETTTLITPDKNNENADGVLINDTVVALGTTNHYRL
TWDLDQYKEDRSAKETIARGFFFVDDYPEEVLDVVENGTSVTTLDGQKVSGITVKTYASLNEAPKYLQDK
FARAKITPTGAFQVFMPDDNQAFYDQYVKTGTSLALLTKMTVKDSLYGQTKTYTNKGYQVDFGNGYETKE
VTNTLVSPEPKKQNLNKDKVDINGKPMLVGTQNHYTLSWDLDQYRGIKADNSQIAQGFYFVDDYPEEALL
PDEAAIQFVTSDGKTVSGITVKAYSQLSEAPKTLQAALSKQKIQPKGAFQVFMPEDPQAFYNSYVTKGEN
ITIVTPMTVLETMINSGKSYENVAYQVDFGQAYETNTVTNFVPKVTPHKSNTNQEGISIDGKTVLPNTVN
YYKIVLDYSQYKDMVVTDDVLAKGFYMVDDYPEEALTLNPDGIQVLDKDGNRVSGISVSTYDSLSEAPKV
VEDAMAKRQFTPKGAIQVLSSDDPKTFYETYVKTGQTLVVTLPMTVKNELTKTGGQYENTAYQIDFGLAY
VTETVVNNVPKLDPQKDVVVDLSHKNNSLDGKEVALNQIINYRLVGAMIPSNRATDLFEYGFEDNYDEKH
DEYNGVYRSYLMTDITLKDGSILKEGTEVTKYTLQQVDTENGLVSISFDKSFLETVSDDSAFQADVYLHM
KRIAAGQVENTYLHTVNGYVISSNTVVTHTPQPEEPSPNQPTPPQPPIETIEPPVPASILPNTGEQESLL
GLIGAGILLGTAYGLKKKEEK

A0A0H3MV42-Surfome

>tr|A0A0H3MV42|A0A0H3MV42_STRS4 Putative glucan-binding surface-anchored protein OS=Streptococcus suis (strain BM407) OX=568814 GN=SSUBM407_0949 PE=4 SV=1
MTKTCNHHFLVNQEKGEKHVFRKSKKYRTLCSVALGTMVTAVVAWGGTVAHADEVVLSVDSTIQRTENPA
TNLPEAQPNPVSEQTESLGSTGKSNGAIAVTVPHETVTQTVEQAKAEGVSTVEDSPMDLGNTTSAAETNQ
QISKAEADAQNQVVAINEVTETYKADKAAYVDEKARIEQENKELSQAYEGANQTGKETNAWVDSKVNDLK
SQYADADVTVKEQVVSSGKGTSVLDYTDYGKAVETIQSTNEQAVADYLTKKTKADDIVAKNQAIQKENEA
GLAKAKADNEAIERRNQAGQAAVDAENRAGQAAVDQANQEKQQLVSDRAVEIEVITKRNQAKEVAARKEN
EAIDAYNAKEMERYQRDLAEISKGEEGYISEALAQALNLNNGEPQAQHGAITRNPNQIISTGDAMLGGYS
RILDSTGFFVYDSFKTGETLSFNYQNLQHARFDGKKISRVTYDITNLVSPAGTDAVKLVVPNDPTEGFIA
YRNDGNGDWRTDKMEFRVVAKYFLEDGSQVTFSKEKPGVFTHSSLNHNDIGLEYVKDSSGKFVPINGSTV
QVTNEGLARSLGSNRASDLNLPEEWDTTSSRYAYKGAIVSTVTSGNTYTVTFGQGDMPQNVGLSYWFALN
TLPVARTVTPYSPKPHVTVELEPVPEPITVTPDVYTPKTFTPEKPVTFTPKPLEEVVQPSLTLTKVTLPV
KPIPKELPTPPQVPTIHYHAYRLTTTPEIMKEVINSDQANLHDKTVAKDSTVIYPLTVDALSPKRTQTTS
LIFEDYLPAGYLFDKETTQKENGNYALSFDESKNFVTLTAKETLLQEVNQDLTKSYQLTAPKLYGTVQND
GATYSNSYKLLVNKGTANSYTVTSNIVTVRTPGDGETTTLITPDKNNENADGVLINDTVVALGTTNHYRL
TWDLDQYKEDRSAKETIARGFFFVDDYPEEVLDVVENGTSVTTLDGQKVSGITVKTYASLNEAPKYLQDK
FARAKITPTGAFQVFMPDDNQAFYDQYVKTGTSLALLTKMTVKDSLYGQTKTYTNKGYQVDFGNGYETKE
VTNTLVSPEPKKQNLNKDKVDINGKPMLVGTQNHYTLSWDLDQYRGIKADNSQIAQGFYFVDDYPEEALL
PDEAAIQFVTSDGKTVSGITVKAYSQLSEAPKTLQAALSKQKIQPKGAFQVFMPEDPQAFYNSYVTKGEN
ITIVTPMTVLETMINSGKSYENVAYQVDFGQAYETNTVTNFVPKVTPHKSNTNQEGISIDGKTVLPNTVN
YYKIVLDYSQYKDMVVTDDVLAKGFYMVDDYPEEALTLNPDGIQVLDKDGNRVSGISVSTYDSLSEAPKV
VEDAMAKRQFTPKGAIQVLSSDDPKTFYETYVKTGQTLVVTLPMTVKNELTKTGGQYENTAYQIDFGLAY
VTETVVNNVPKLDPQKDVVVDLSHKNNSLDGKEVALNQIINYRLVGAMIPSNRATDLFEYGFEDNYDEKH
DEYNGVYRSYLMTDITLKDGSILKEGTEVTKYTLQQVDTENGLVSISFDKSFLETVSDDSAFQADVYLHM
KRIAAGQVENTYLHTVNGYVISSNTVVTHTPQPEEPSPNQPTPPQPPIETIEPPVPASILPNTGEQESLL
GLIGAGILLGTAYGLKKKEEK

A0A0H3MVU2-Exoproteome

```
>tr|A0A0H3MVU2|A0A0H3MVU2_STRS4 Beta-N-acetylhexosaminidase
OS=Streptococcus suis (strain BM407) OX=568814 GN=SSUBM407_1236 PE=3
SV=1
MKKETSKFTKKVILGQLLICTSFLVWAGQTVAAEEVSQGEPVAINNETPPVIEAKPLVEATPSTETEPST
PEEKQEEPQVAGKNVVEGKKPVTNSQHQMVSELSTVPSVSIKNPDAATDGSAYGSSDDANSNTKIIAGKE
TGEPDNGYSKWDHVYVQYDFEKEVPVHTVKIHRNTYDNAVSTFKDVKVELSTSADFSPETTEVIFKQADV
EETIANKGQPQVIQLPAPINARYIRIWGRGHYIQNTNSSWKGYSNGVLFNEIEVLAQVEADAGTNEEPQT
ELVNLAKLKKPYFYGKAPTNPEAINDGKFDENYTTQESIGDKAYLQYEFRNVYTIEEIKVQLAPGEYRNF
RIDLDDDYNDGNNYNTIYKNENFTIDKEQIITIKTPKNSKARYVRFRGTRVDDTPLRYSEIQIMGRGKSY
DESAPEYVPPKSEYDTLVWSDEFNGDKIDESKWQIIDGMWNHAAIYNRKAVSIKKDGDKSYLSIRSTNHK
DKKTLVEAVGHDRYDDKELPDKVTWSSGRLESKDKYSFQFGRMAVRAKVNDSKGIWPAIWMLAQDETGHD
EIDVLEYLGQNPWYAWTTNHFGILAKNKKSDGSAYKNYEAWSQEFHVYEVEWTPDVIKWYIDGKFVFQTD
RGKDRDGMHTRPMFPILETQVGDGWVGDVDYKRNMTKQDSEYLVDWVRIYQKADQSKVRFDNLDDAKPND
YVIKPQQKLGHLVTVSNGKAAHENKNNFYYGGQPRYETSRLYADGDGENALVYKIANPEALHLTTYYKTL
EDYSVYNKEAGANEGKSVRKHLINAKDGEIDFTVYSSVDGKSWNKENVTVVDNFVEATPAYARTTFDIRN
IRKGTKFVKIVFPTVKGISYKLEDGTEKGLQASDVQLAKVTFVATHPEAEPEKPIVDQEKLALSKVFSLD
AGRKYFSVNQIKEIIDLISSYGYTDMNLLLGNDGLRFLLDDMNLTVGDKVYDSEAVRKAIINGNKHYYDD
PNGTTLSQSEMDDILAYAKEKNISIIPTINSPGHMDAIVEAMEELGIKSPKFSYNGKTSARTVDLKNDQA
VAFTKALIDKYASYFAGKSKIFNIGLDEYANDATNASGWHILQTQGDYGKFIQYANDLAAIVKKHGLEPM
AFNDGIYYNHDTSSGTFDKDIIASFWTGGWGGYDVASSKFLHEKGHKILNTNDAWYYVIGREAEGLGWYN
LDQGLRGTKSTRFDQVPKSENTKVPIIGSMVAVWADDPSKPYSYDSIKKLLHSFTENNSDYFLAEYRDLR
EEVGKIPTDLNSYTPESVATLKEVLNKIDWKLSRNNQAFVDGYLAEVKAAREALVPVTAKGDSVKAEPLP
EGKLPLITAKGKSVQAEPRPAFKGYLTSNYGPSVTPLSLVSKPSSEQSNSKQAASQKKELPNTGVVDGLG
FSLLGMIGLAFASRRRKKE
```

A0A0H3MVU2-Surfome

```
>tr|A0A0H3MVU2|A0A0H3MVU2_STRS4 Beta-N-acetylhexosaminidase
OS=Streptococcus suis (strain BM407) OX=568814 GN=SSUBM407_1236 PE=3
SV=1
MKKETSKFTKKVILGQLLICTSFLVWAGQTVAAEEVSQGEPVAINNETPPVIEAKPLVEATPSTETEPST
PEEKQEEPQVAGKNVVEGKKPVTNSQHQMVSELSTVPSVSIKNPDAATDGSAYGSSDDANSNTKIIAGKE
TGEPDNGYSKWDHVYVQYDFEKEVPVHTVKIHRNTYDNAVSTFKDVKVELSTSADFSPETTEVIFKQADV
EETIANKGQPQVIQLPAPINARYIRIWGRGHYIQNTNSSWKGYSNGVLFNEIEVLAQVEADAGTNEEPQT
ELVNLAKLKKPYFYGKAPTNPEAINDGKFDENYTTQESIGDKAYLQYEFRNVYTIEEIKVQLAPGEYRNF
RIDLDDDYNDGNNYNTIYKNENFTIDKEQIITIKTPKNSKARYVRFRGTRVDDTPLRYSEIQIMGRGKSY
DESAPEYVPPKSEYDTLVWSDEFNGDKIDESKWQIIDGMWNHAAIYNRKAVSIKKDGDKSYLSIRSTNHK
DKKTLVEAVGHDRYDDKELPDKVTWSSGRLESKDKYSFQFGRMAVRAKVNDSKGIWPAIWMLAQDETGHD
EIDVLEYLGQNPWYAWTTNHFGILAKNKKSDGSAYKNYEAWSQEFHVYEVEWTPDVIKWYIDGKFVFQTD
RGKDRDGMHTRPMFPILETQVGDGWVGDVDYKRNMTKQDSEYLVDWVRIYQKADQSKVRFDNLDDAKPND
YVIKPQQKLGHLVTVSNGKAAHENKNNFYYGGQPRYETSRLYADGDGENALVYKIANPEALHLTTYYKTL
EDYSVYNKEAGANEGKSVRKHLINAKDGEIDFTVYSSVDGKSWNKENVTVVDNFVEATPAYARTTFDIRN
IRKGTKFVKIVFPTVKGISYKLEDGTEKGLQASDVQLAKVTFVATHPEAEPEKPIVDQEKLALSKVFSLD
AGRKYFSVNQIKEIIDLISSYGYTDMNLLLGNDGLRFLLDDMNLTVGDKVYDSEAVRKAIINGNKHYYDD
PNGTTLSQSEMDDILAYAKEKNISIIPTINSPGHMDAIVEAMEELGIKSPKFSYNGKTSARTVDLKNDQA
VAFTKALIDKYASYFAGKSKIFNIGLDEYANDATNASGWHILQTQGDYGKFIQYANDLAAIVKKHGLEPM
AFNDGIYYNHDTSSGTFDKDIIASFWTGGWGGYDVASSKFLHEKGHKILNTNDAWYYVIGREAEGLGWYN
LDQGLRGTKSTRFDQVPKSENTKVPIIGSMVAVWADDPSKPYSYDSIKKLLHSFTENNSDYFLAEYRDLR
EEVGKIPTDLNSYTPESVATLKEVLNKIDWKLSRNNQAFVDGYLAEVKAAREALVPVTAKGDSVKAEPLP
EGKLPLITAKGKSVQAEPRPAFKGYLTSNYGPSVTPLSLVSKPSSEQSNSKQAASQKKELPNTGVVDGLG
FSLLGMIGLAFASRRRKKE
```

This protein was not found in the surfome analysis

A0A0H3N5E4-Exoproteome

>tr|A0A0H3N5E4|A0A0H3N5E4_STRS4 Putative surface-anchored 5'-
nucleotidase OS=Streptococcus suis (strain BM407) OX=568814
GN=SSUBM407_1432 PE=3 SV=1
MKKNIRLKSSILALVAGFSVIATQAVLADELAVQIMGVNDFHGALDMTGTARLEGETVRNAGTAALLDAY
MDDSQAEFEETAAETETPAESIRVQAGDMVGASPSNSGLLQDEPTVKVFNKMDVEYGTLGNHEFDEGLDE
YNRIMTGEAPKKGQFNEIVDNYTREAAKQEIVIANVIDKETGEIPYGWKPYAIKTIPVNDKEAKIGFIGV
VTTEIPNLVLKKNYEQYTFLNEAETIAKYARELAEKGVNAIVVLAHVPATSKDGVAAGEAADMIAKLNEI
YPEHSVDLVFAGHNHVYTNGTTGKTLIVQATSQGKAYADVRAVYDTDIADFKAVPTAKIIAVAPGQKTPS
PEIQAIVDEANTIVKKVTEQKIATASQATDISREVNEFKESAVGNLVTSAQLAIAKKSGYDVDFAMTNDG
GIRADLKVQEDGTVTWGAAQAVQPFGNILQVVQMTGEQIYTALNQQYDEGEKYFLQMSGIKYIYTKADNP
TEENPYKVVKAFKEDGTEIVPTETYTLVINDFLFGGGDGFSIFKEAKLIGAINPDTEVFVEYLTDLEKAG
QTISATIPGRKAFVEKYVEEPKAEEKEDNAGTTTDVKTPEKANDGGDSVTNQKATEQPAPSGSMAPISNK
KTEKASGNQTLPNTGQEALGSLLISLGGLVSLGMAVSVRRKEGE

A0A0H3N5E4-Surfome

>tr|A0A0H3N5E4|A0A0H3N5E4_STRS4 Putative surface-anchored 5'-
nucleotidase OS=Streptococcus suis (strain BM407) OX=568814
GN=SSUBM407_1432 PE=3 SV=1
MKKNIRLKSSILALVAGFSVIATQAVLADELAVQIMGVNDFHGALDMTGTARLEGETVRNAGTAALLDAY
MDDSQAEFEETAAETETPAESIRVQAGDMVGASPSNSGLLQDEPTVKVFNKMDVEYGTLGNHEFDEGLDE
YNRIMTGEAPKKGQFNEIVDNYTREAAKQEIVIANVIDKETGEIPYGWKPYAIKTIPVNDKEAKIGFIGV
VTTEIPNLVLKKNYEQYTFLNEAETIAKYARELAEKGVNAIVVLAHVPATSKDGVAAGEAADMIAKLNEI
YPEHSVDLVFAGHNHVYTNGTTGKTLIVQATSQGKAYADVRAVYDTDIADFKAVPTAKIIAVAPGQKTPS
PEIQAIVDEANTIVKKVTEQKIATASQATDISREVNEFKESAVGNLVTSAQLAIAKKSGYDVDFAMTNDG
GIRADLKVQEDGTVTWGAAQAVQPFGNILQVVQMTGEQIYTALNQQYDEGEKYFLQMSGIKYIYTKADNP
TEENPYKVVKAFKEDGTEIVPTETYTLVINDFLFGGGDGFSIFKEAKLIGAINPDTEVFVEYLTDLEKAG
QTISATIPGRKAFVEKYVEEPKAEEKEDNAGTTTDVKTPEKANDGGDSVTNQKATEQPAPSGSMAPISNK
KTEKASGNQTLPNTGQEALGSLLISLGGLVSLGMAVSVRRKEGE

A0A0H3N5P5-Exoproteome

>tr|A0A0H3N5P5|A0A0H3N5P5_STRS4 Putative surface-anchored protein OS=Streptococcus suis (strain BM407) OX=568814 GN=SSUBM407_1550 PE=4 SV=1
MNSKIFSLRKSKMGLVSVAIAFLWIGTGMNMETAMAEETDATALETQLESTESSLTNTVSENAEAEEVTD
EVPSEEKKSEEMEDMEEELSFIENHLEVAPIQAGDQTISGNTTPGGYVAITIDGEAITSIENILEADDKG
DFSYRLSKPLAHSQTVEISALPKQFWTLEADSEERKVVVRTNRHPEAYEIPAKRLEKTSNGMHQVFIEPV
FEHTSKVIGHTSVKGSVYLSINGSFVSDKTLIDPKDGRFEVTFSESLAGSKFKADDRLVLSFVSEDGQPV
ITNTIVKPLVKEKVSSQMTVKPLSSATSVLEGTTFPLGRVHLYNADTSEFIMEAIADETGHYKIALPALQ
SEDKYYRLTHNQQEDLVSVHLDTVDGSSILLDKSVMASLATYLQDADMDEATDEDPIIVPKLHNKKDYIV
GRTIHLNAYVRMVSSIKGKQYPPVQVDELGFFGFQIQDLQLPFEKGERIRFEIIDPVTNNIIASKEEVVG
QYLEDEDVMDLPFQVEKVTTDHGYISGKTAPDVMIELVSTQNGEEIIGKTSTDSTGRFEFDLGSRVLKNG
ETLSFRAFDKEGEQVAWEVVTVQKGNGHRINKPDKKDEKEEQPSKEITKNIEQSNTLEQTTLPPVRQTLT
DKKVEQNAEPSKEETVSIFDDSKKDMPTKQEKMARTVRDKGTKGNVSVHDSGENTQVQSLPKTGEKTSLV
ANIMLSIILFLFALFIGKKKITESE

A0A0H3N5P5- Surfome

>tr|A0A0H3N5P5|A0A0H3N5P5_STRS4 Putative surface-anchored protein OS=Streptococcus suis (strain BM407) OX=568814 GN=SSUBM407_1550 PE=4 SV=1
MNSKIFSLRKSKMGLVSVAIAFLWIGTGMNMETAMAEETDATALETQLESTESSLTNTVSENAEAEEVTD
EVPSEEKKSEEMEDMEEELSFIENHLEVAPIQAGDQTISGNTTPGGYVAITIDGEAITSIENILEADDKG
DFSYRLSKPLAHSQTVEISALPKQFWTLEADSEERKVVVRTNRHPEAYEIPAKRLEKTSNGMHQVFIEPV
FEHTSKVIGHTSVKGSVYLSINGSFVSDKTLIDPKDGRFEVTFSESLAGSKFKADDRLVLSFVSEDGQPV
ITNTIVKPLVKEKVSSQMTVKPLSSATSVLEGTTFPLGRVHLYNADTSEFIMEAIADETGHYKIALPALQ
SEDKYYRLTHNQQEDLVSVHLDTVDGSSILLDKSVMASLATYLQDADMDEATDEDPIIVPKLHNKKDYIV
GRTIHLNAYVRMVSSIKGKQYPPVQVDELGFFGFQIQDLQLPFEKGERIRFEIIDPVTNNIIASKEEVVG
QYLEDEDVMDLPFQVEKVTTDHGYISGKTAPDVMIELVSTQNGEEIIGKTSTDSTGRFEFDLGSRVLKNG
ETLSFRAFDKEGEQVAWEVVTVQKGNGHRINKPDKKDEKEEQPSKEITKNIEQSNTLEQTTLPPVRQTLT
DKKVEQNAEPSKEETVSIFDDSKKDMPTKQEKMARTVRDKGTKGNVSVHDSGENTQVQSLPKTGEKTSLV
ANIMLSIILFLFALFIGKKKITESE

A0A0H3MXZ4-Exoproteome

>tr|A0A0H3MXZ4|A0A0H3MXZ4_STRS4 Mannosyl-glycoprotein endo-beta-N-acetylglucosaminidase OS=Streptococcus suis (strain BM407) OX=568814 GN=SSUBM407_1786 PE=3 SV=1
MTKCDFDKRQRFGIRKFTIGACSVLIGATLFGTQVSADQLENVDSLSEPAVELSSVVNEHVEKTEAEEGM
SPISADEKAVTEELNGEASVSDSREEKLPSAPSDNPENKLEDTEEAEDKEVDSSDSGLLLSDFPQVEDQG
IRPKEIKFDNWDQVLAWEPGARPDDDLNRASVELVERFRGHVVNERANENAKVEALSNTNSKAKDHASVG
GEEFKAYAFDYWQYIDSMVFWEGLIPSPDVIDAGHRNGVPVLGTIFYNWSSSIEDQEKFVSSMRQDPDGS
FPVARKLVDMAKYYGFDGYFINQETTGGLVQPLGETMRNFMLYTKEYAESIGYPITYSWYDAMTYEYGRY
HEDGLGEYNYPFMEKEGDKVPADHFFANFNWTRAKNNYSIEVAKRIGRNPFDIFAGFELQRGGSYKTQIN
WDALFDENGKLKLSLGLFAPDTITSLGATGEDYHENENIFFTGYQGDPTKQKPSDKNWYGLANLIADKTA
ITSSNFNSSFNTGHGKKWFVDGKVAKDGEWNYRSVSGILPTWRWWIEASGEKLAAAYDFDDAYNGGNSLR
FTGDLEKDGKQEIKLYSTKIPLSETSRLRVAHKGGQGANISLALALEPDYQFANKEAWRLLKLSGDWQDQ
TFDLSSLAGKTVYGIQVVVENEAALSDFDFRLGQLAIYDQENSPTAPKDGQVLAKRLKNAQDAEAVISFT
GTDDADYYEVYAQVDGQWKLLTGSSNTRIYLPQLVRSAQAEGRTQALKVHAVGKNGLRSEAGEFIFDWEM
TVKDTSLPKPSAENIVLGAEVIGSSFAKKEGGEGIEGMLNGTITSLSDKWSSHQLSGHVDIRLTQPRTVV
RWAMDHAGAGGESVNDGLMNTKDFDLYYKNENGDWVLAKEVRGNRDHVTDIVLDKPIRAQEWRLDVLTSD
NGTPWKAIRIYNWRMYEELDMETPNIPMTHAVARHLGNHQIQVGFKDVPANRKIALYSSPDAVKPLAELE
TTESGNLIFDPIRFESLPEFIYYRTLEEGKDWSNLLAIRVPQGEKVVKAMEWIAPLEKKVYRQGSPLSLA
GAQFRVVFEGDWPAERVNTTNPAVTVRGFDPQKLGEQTLTVSYLGETLAQPLMVYVVEDIAQGEKKAVGL
EIQQLPKVQYVVGDDLDVTNGRFAIIYDDESTQSFSLTEAGVEIVGFDSRKEGRQSLGLRYAGLETSFDV
LVSPKPIVNDEYLKQKIAEIEALQTQTAYLYSSEQTQLALQTALEEAKSVVADTNRTVEQVEAAQTLLEK
QLGELDGEKLYQADLGRLNQLIQEVKGIDRPINSSELSSLLEKTEKILGSDSIRPDEMKELLSEWTDLFE
QSSLVTHVGTRDSMESKNLLAPEKPRMDLTYELVPRQIITRTSTDLPLGKERIVQEGADGQMMIAHLVYS
DGRRELYSRIVADESQPKIVEVGSGLPIQQEKTLMVENSLQVEKQIRDASLQKSGLPQTGDTYQEKYVFL
GLIGVALAGLSQLAKLRKQGE

A0A0H3MXZ4-Surfome

>tr|A0A0H3MXZ4|A0A0H3MXZ4_STRS4 Mannosyl-glycoprotein endo-beta-N-acetylglucosaminidase OS=Streptococcus suis (strain BM407) OX=568814 GN=SSUBM407_1786 PE=3 SV=1
MTKCDFDKRQRFGIRKFTIGACSVLIGATLFGTQVSADQLENVDSLSEPAVELSSVVNEHVEKTEAEEGM
SPISADEKAVTEELNGEASVSDSREEKLPSAPSDNPENKLEDTEEAEDKEVDSSDSGLLLSDFPQVEDQG
IRPKEIKFDNWDQVLAWEPGARPDDDLNRASVELVERFRGHVVNERANENAKVEALSNTNSKAKDHASVG
GEEFKAYAFDYWQYIDSMVFWEGLIPSPDVIDAGHRNGVPVLGTIFYNWSSSIEDQEKFVSSMRQDPDGS
FPVARKLVDMAKYYGFDGYFINQETTGGLVQPLGETMRNFMLYTKEYAESIGYPITYSWYDAMTYEYGRY
HEDGLGEYNYPFMEKEGDKVPADHFFANFNWTRAKNNYSIEVAKRIGRNPFDIFAGFELQRGGSYKTQIN
WDALFDENGKLKLSLGLFAPDTITSLGATGEDYHENENIFFTGYQGDPTKQKPSDKNWYGLANLIADKTA
ITSSNFNSSFNTGHGKKWFVDGKVAKDGEWNYRSVSGILPTWRWWIEASGEKLAAAYDFDDAYNGGNSLR
FTGDLEKDGKQEIKLYSTKIPLSETSRLRVAHKGGQGANISLALALEPDYQFANKEAWRLLKLSGDWQDQ
TFDLSSLAGKTVYGIQVVVENEAALSDFDFRLGQLAIYDQENSPTAPKDGQVLAKRLKNAQDAEAVISFT
GTDDADYYEVYAQVDGQWKLLTGSSNTRIYLPQLVRSAQAEGRTQALKVHAVGKNGLRSEAGEFIFDWEM
TVKDTSLPKPSAENIVLGAEVIGSSFAKKEGGEGIEGMLNGTITSLSDKWSSHQLSGHVDIRLTQPRTVV
RWAMDHAGAGGESVNDGLMNTKDFDLYYKNENGDWVLAKEVRGNRDHVTDIVLDKPIRAQEWRLDVLTSD
NGTPWKAIRIYNWRMYEELDMETPNIPMTHAVARHLGNHQIQVGFKDVPANRKIALYSSPDAVKPLAELE
TTESGNLIFDPIRFESLPEFIYYRTLEEGKDWSNLLAIRVPQGEKVVKAMEWIAPLEKKVYRQGSPLSLA
GAQFRVVFEGDWPAERVNTTNPAVTVRGFDPQKLGEQTLTVSYLGETLAQPLMVYVVEDIAQGEKKAVGL
EIQQLPKVQYVVGDDLDVTNGRFAIIYDDESTQSFSLTEAGVEIVGFDSRKEGRQSLGLRYAGLETSFDV
LVSPKPIVNDEYLKQKIAEIEALQTQTAYLYSSEQTQLALQTALEEAKSVVADTNRTVEQVEAAQTLLEK
QLGELDGEKLYQADLGRLNQLIQEVKGIDRPINSSELSSLLEKTEKILGSDSIRPDEMKELLSEWTDLFE
QSSLVTHVGTRDSMESKNLLAPEKPRMDLTYELVPRQIITRTSTDLPLGKERIVQEGADGQMMIAHLVYS
DGRRELYSRIVADESQPKIVEVGSGLPIQQEKTLMVENSLQVEKQIRDASLQKSGLPQTGDTYQEKYVFL
GLIGVALAGLSQLAKLRKQGE
This protein was not found in the surfome analysis

A0A0H3MXF6-Exoproteome

>tr|A0A0H3MXF6|A0A0H3MXF6_STRS4 Surface-anchored DNA nuclease
OS=Streptococcus suis (strain BM407) OX=568814 GN=ssnA PE=4 SV=1
MKIRNRSLFYTVGSVAVTAGLLLSLATSPIPSVHATEVAIENYPSLAITKSEVTIQGYLIAPLNSSGTAF
DATNKTNLALGASMDTAAADTIPIQLKEPLRSQFNLVNHPELVGKLVRITGTSDTYMKRAGIKPATAIEI
VDSSSTNVQPPTSENTATKPSDLVSTPIATVRSGAQGTEYTVSGKIISLVNGWGGNGFYLQGSDGAGIYI
YPGAALGYQLGDTVQLTGTLGEYKGELQLTTVSNHKAISENFNTPITETNIAQLATQAQATLVSLKNLTV
GDIQSDSYQNSTFTVTDSEGQTVDVRLDSRTGIKTADLLNRINKGDKINLTAILSTYNGKIQLKPFDLSH
FEVIEKATTEAGLGKTEAVTVGRIQGASHQSPLVNQSVMLKNVVVTYVTSANNFYVQDVTPDGDTKTSDG
INIFTDKLKTNVKVGDLVTIAGRVEEYQGRGYAERDKTDLTITQIRATEVTVDGTAPVPSPIVLGLDRTI
PADIIDNDGLAQFDPEQDALDFWESVEGMVVAVDDAKILGPLKNKEIYVTPATSQLPLNNVGGVNLRPEG
NNTNIIPLLLKNGKQIVKSGDYFIGRIAGPVTYSYTNYKVYVDDSTLPTLHEGATKPETTTIIPNDDKLT
IASYNIENFSANSKSTSDAKVQRIAKSFVSDLHSPDVIGLIEVQDNNGATNDGTTDASKSAERLIAAIQA
AGGPTYTYVDIAPENNKDGGQEGGNIRVGFLYNSKRVSLSDKPIGTATQAVAWENGELNLSLGRIDPTNP
AWAAVRKTLAAEFVFKGEKVVVLANHLNSKRGDNGLYGKIQPVSFKSEEKRHILAQTIADFTKAGLAQNP
NANIVMLGDFNDYEFTKTIEILEAGGMANLVSRHDASDRFSYFYNGNNQSLDNMLVSTNLFERYAFDMVH
VNSAFMEEHGRASDHDPLLVQLDVTKAQEPTQPEPSDKQTDDSGTVNNSDDNGTTNNNKPTNLSTSNQTA
VNADDRSGATDKRQTTVTPTANNSQKKILPKTGGETSFVLITIGLVFLSACLVKKQKES

A0A0H3MXF6-Surfome

>tr|A0A0H3MXF6|A0A0H3MXF6_STRS4 Surface-anchored DNA nuclease
OS=Streptococcus suis (strain BM407) OX=568814 GN=ssnA PE=4 SV=1
MKIRNRSLFYTVGSVAVTAGLLLSLATSPIPSVHATEVAIENYPSLAITKSEVTIQGYLIAPLNSSGTAF
DATNKTNLALGASMDTAAADTIPIQLKEPLRSQFNLVNHPELVGKLVRITGTSDTYMKRAGIKPATAIEI
VDSSSTNVQPPTSENTATKPSDLVSTPIATVRSGAQGTEYTVSGKIISLVNGWGGNGFYLQGSDGAGIYI
YPGAALGYQLGDTVQLTGTLGEYKGELQLTTVSNHKAISENFNTPITETNIAQLATQAQATLVSLKNLTV
GDIQSDSYQNSTFTVTDSEGQTVDVRLDSRTGIKTADLLNRINKGDKINLTAILSTYNGKIQLKPFDLSH
FEVIEKATTEAGLGKTEAVTVGRIQGASHQSPLVNQSVMLKNVVVTYVTSANNFYVQDVTPDGDTKTSDG
INIFTDKLKTNVKVGDLVTIAGRVEEYQGRGYAERDKTDLTITQIRATEVTVDGTAPVPSPIVLGLDRTI
PADIIDNDGLAQFDPEQDALDFWESVEGMVVAVDDAKILGPLKNKEIYVTPATSQLPLNNVGGVNLRPEG
NNTNIIPLLLKNGKQIVKSGDYFIGRIAGPVTYSYTNYKVYVDDSTLPTLHEGATKPETTTIIPNDDKLT
IASYNIENFSANSKSTSDAKVQRIAKSFVSDLHSPDVIGLIEVQDNNGATNDGTTDASKSAERLIAAIQA
AGGPTYTYVDIAPENNKDGGQEGGNIRVGFLYNSKRVSLSDKPIGTATQAVAWENGELNLSLGRIDPTNP
AWAAVRKTLAAEFVFKGEKVVVLANHLNSKRGDNGLYGKIQPVSFKSEEKRHILAQTIADFTKAGLAQNP
NANIVMLGDFNDYEFTKTIEILEAGGMANLVSRHDASDRFSYFYNGNNQSLDNMLVSTNLFERYAFDMVH
VNSAFMEEHGRASDHDPLLVQLDVTKAQEPTQPEPSDKQTDDSGTVNNSDDNGTTNNNKPTNLSTSNQTA
VNADDRSGATDKRQTTVTPTANNSQKKILPKTGGETSFVLITIGLVFLSACLVKKQKES

A0A0H3N6J4-Exoproteome

```
>tr|A0A0H3N6J4|A0A0H3N6J4_STRS4 Putative surface-anchored serine
protease OS=Streptococcus suis (strain BM407) OX=568814
GN=SSUBM407_1843 PE=3 SV=1
MKQKWSQIENKQRFSIKKLSVGVASVSIGFFITGVPMVQADTSGEGLESTVAVATDMDSRQNSAVEKKED
GPLSDDPVKTEQVDEPVAEEGVVEEVVDTEAGEESGLLTDQAATEIETTAGKTTDESKEKEDISGKEASA
PQTIPQESQLEPEEVTTGRYILQFSEENRNLVLDKLKKIDGVKIVHEYKEVLTGASVEVGKESLSDVKAI
TELTSLEESRRIRPTLHTAKQLVGALKASSKYQTDGRGMVIAVIDSGLDIKHKDMRLDDGVIPKIKDITP
STTGTYTLKVPHGYNYVSGNDNLYDDTHEPHGMHIAGTLAGNATDEEVASKKGVDGIAPNAQLLVYKIFS
NDPKNYKAETEDAAYAAIEDAIKHGADVISLSVGYYDSGLPGNAYYTIAKRAAEKGIIITAAIGNAGASS
SDTSFDLHTNNALGAVDTATTVGVAATPAVIAVGSARNTHLVQREFMLNGQSFGYYPIGYTTLTEGKYEF
VDAGNGHWEEVQGLDLAGKVAVIKKDKFDLKDAVRNLKFKDVAGIIVINTDQGWNKDYYRTHQLLVDDKT
LLSYSSIWGISLSGEDGRRLLEVANQSQGNTGLVLKPTIGMKKLIEVPTVSGFSSWGPTVNLELKPEIVA
PGEDVYATLNDNRYGSMSGTSMASPIVAGASALLLPRIRQMTPPEGMTRMDLLRIILMNTATPLVDVLDS
SGHALENSPRQQGAGLLQIDRAFETDVILHHRLKGGVELKEIGRETEFEVTLENLGNQQRSFAISAGKVL
TSQDVPVDRIGRSGKVVKEIHATEIKGSSIHLSEQSIQLGPKEKRTIRLKLDAGEAKDQFAEGYIYFKSL
TEGQSDISIPYFGFVGDWSKERIVDAPAWETSSKLKLTSVLSSYKHNKSGRYIELGREKIQDNQSPLNPD
NIAIQNQHSDSQIGNAFVRFALLRDITNYDLDIVKEATEDAPVLRRIDTGTMLSRVRYVDYFESLSEYSK
LRTPIELHRWDGKVYDASNDENIPAPEGQYFFRLRVKNKENGAYQYTYLPVKIDNQKPEIVAIDTNRLSS
HRELVVTAKDNNKVWEVRANLNGEDLLVEKVVDDAGQLHYHLKEVELPLDAKNHLRVEVMDIAGNVVAVE
KDLMAPVIQFKNLEDLMAIRSKKTVEIKANVSAQVSDVQANLDAQAVNYSLENGQLSLQIPEQSDGRHSF
ELILKDKDGNLIYTKTLNYLVDNEKPTIDLDIEKDEEDEEVIQIGKNGRFTLKGKVSDNVSLPKDIKLYY
SNLDIGKGERKIIDVKEDGSFEQDFFKSDFPRAIMLTAVDEKGNKLKDLRINTSPESLDEEEETEVPITV
NNWLIDPIRFNKESLGRELDSGLVDFKKQEDGTYLFTFEIEAETEQAHSVRINGGEKRYFEDGKLTYPVT
LIEEGNVVDISVYNEADELTYTKKYQMLVDTENPVLQLENEVLPLERQVVDSEEDEDEENQYAGVLLADA
DGHLTLTGSAKDNGIYWSLKINEDFVARGGFWRQYGNNEKAFRYELHSLKDGDTVKLDLSDSFGNAVVKK
YKVRLNDKEVSEQVPEKDLHVERSDKDQTPSIPILKSEAHIPMPKEENSLAPQTGSTEIALLTGDTREDG
VEHLGKLTKHEEPLGISDERIEVSVPHREFFERSGIGETGALAADTSGKLPQTGDSLGSVFISTLLGLFG
GAMALGNLKRKE
```

A0A0H3N6J4-Surfome

>tr|A0A0H3N6J4|A0A0H3N6J4_STRS4 Putative surface-anchored serine protease OS=Streptococcus suis (strain BM407) OX=568814 GN=SSUBM407_1843 PE=3 SV=1

```
MKQKWSQIENKQRFSIKKLSVGVASVSIGFFITGVPMVQADTSGEGLESTVAVATDMDSRQNSAVEKKED
GPLSDDPVKTEQVDEPVAEEGVVEEVVDTEAGEESGLLTDQAATEIETTAGKTTDESKEKEDISGKEASA
PQTIPQESQLEPEEVTTGRYILQFSEENRNLVLDKLKKIDGVKIVHEYKEVLTGASVEVGKESLSDVKAI
TELTSLEESRRIRPTLHTAKQLVGALKASSKYQTDGRGMVIAVIDSGLDIKHKDMRLDDGVIPKIKDITP
STTGTYTLKVPHGYNYVSGNDNLYDDTHEPHGMHIAGTLAGNATDEEVASKKGVDGIAPNAQLLVYKIFS
NDPKNYKAETEDAAYAAIEDAIKHGADVISLSVGYYDSGLPGNAYYTIAKRAAEKGIIITAAIGNAGASS
SDTSFDLHTNNALGAVDTATTVGVAATPAVIAVGSARNTHLVQREFMLNGQSFGYYPIGYTTLTEGKYEF
VDAGNGHWEEVQGLDLAGKVAVIKKDKFDLKDAVRNLKFKDVAGIIVINTDQGWNKDYYRTHQLLVDDKT
LLSYSSIWGISLSGEDGRRLLEVANQSQGNTGLVLKPTIGMKKLIEVPTVSGFSSWGPTVNLELKPEIVA
PGEDVYATLNDNRYGSMSGTSMASPIVAGASALLLPRIRQMTPPEGMTRMDLLRIILMNTATPLVDVLDS
SGHALENSPRQQGAGLLQIDRAFETDVILHHRLKGGVELKEIGRETEFEVTLENLGNQQRSFAISAGKVL
TSQDVPVDRIGRSGKVVKEIHATEIKGSSIHLSEQSIQLGPKEKRTIRLKLDAGEAKDQFAEGYIYFKSL
TEGQSDISIPYFGFVGDWSKERIVDAPAWETSSKLKLTSVLSSYKHNKSGRYIELGREKIQDNQSPLNPD
NIAIQNQHSDSQIGNAFVRFALLRDITNYDLDIVKEATEDAPVLRRIDTGTMLSRVRYVDYFESLSEYSK
LRTPIELHRWDGKVYDASNDENIPAPEGQYFFRLRVKNKENGAYQYTYLPVKIDNQKPEIVAIDTNRLSS
HRELVVTAKDNNKVWEVRANLNGEDLLVEKVVDDAGQLHYHLKEVELPLDAKNHLRVEVMDIAGNVVAVE
KDLMAPVIQFKNLEDLMAIRSKKTVEIKANVSAQVSDVQANLDAQAVNYSLENGQLSLQIPEQSDGRHSF
ELILKDKDGNLIYTKTLNYLVDNEKPTIDLDIEKDEEDEEVIQIGKNGRFTLKGKVSDNVSLPKDIKLYY
SNLDIGKGERKIIDVKEDGSFEQDFFKSDFPRAIMLTAVDEKGNKLKDLRINTSPESLDEEEETEVPITV
NNWLIDPIRFNKESLGRELDSGLVDFKKQEDGTYLFTFEIEAETEQAHSVRINGGEKRYFEDGKLTYPVT
LIEEGNVVDISVYNEADELTYTKKYQMLVDTENPVLQLENEVLPLERQVVDSEEDEDEENQYAGVLLADA
DGHLTLTGSAKDNGIYWSLKINEDFVARGGFWRQYGNNEKAFRYELHSLKDGDTVKLDLSDSFGNAVVKK
YKVRLNDKEVSEQVPEKDLHVERSDKDQTPSIPILKSEAHIPMPKEENSLAPQTGSTEIALLTGDTREDG
VEHLGKLTKHEEPLGISDERIEVSVPHREFFERSGIGETGALAADTSGKLPQTGDSLGSVFISTLLGLFG
GAMALGNLKRKE
```

A0A0H3MYG2-Exoproteome

>tr|A0A0H3MYG2|A0A0H3MYG2_STRS4 Putative surface-anchored amylopullulanase OS=Streptococcus suis (strain BM407) OX=568814 GN=SSUBM407_1919 PE=3 SV=1
MKRTGMLEKKQIFGIRKLNVGVASVGIAAALFLSGAGQLVQAEEVVLPASTSSTTLSEQDGGAVEATTTA
IEATDSMVAEATAVTETADATAPEEAPIEEGSIRLHFENVDETAPESQGLWTWGGVAEPSDGNQWPTDTA
NFSSSQVDDYGHYVDIKKSETPGTIGYLVLKNGEKITESDQKVELLVPEQNEAWIASDYSVSSYEPLKDE
NVLRINYTREDNNYEGWGVWTWGDTTEASSGWPAGAVDFKLGKYGAYVDIPLSNGLDSKLGFLLINQNNP
DLAGNKTIDLAFADRKRHSQIFLRNDDDKVYTNPYFIEEKVELDTSKATPGTKNVTVGASVKAPFNYNES
GLVSVTVTNPENAEIVKMEVDTSAIGGGLVPISTELNRVTIKATSSTAPGTYSLPVKVYDKDNGYYETKL
DVTITERIKADGEKDWDEQVIYFMMTDRFYNGDTGNDKLVEGTASNPRGLYQGGDFKGVTAKLDYLKELG
VDSIWLTPIVENIPQNVGSVKDGEYYAYHGYWASNFEKLNPHLGSLADFHELIDAAAEKGINIIVDVVLN
HAGYGTEETFAGMVRTKEEDKQGDDQLGSLSGLPDFKTEEAAVRNQLVAWQASWLERSTTAKGNSIYAFR
VDTVKHVDDTTWQHFKNELVDRDPDFHLIGETWGANYKDTKGDLGIGTMDSLLDFGFKDIAKYLVNGQLK
AAGKELEERSKVLTSAASLGQFLGSHDEDGFLYSLGGAEKEGNLDKLKLAASLLITAKGQPVIYYGEEVG
QSGQNNWPVYDNRYDFDWSKVETSDITDHYQKLLAFRNANSTLLSRGDTSTLAGNDSQGWLISKRSYQDQ
AAYLVFSTNTENKEMALEVSGKDVVVTDAYTGKSYQAIEKDGKWVVQIELPTIGQGGTMLLQTEAGDIVN
ASVQGATEEPIEAGYFRVHFKTLPSDNLSSLGLWTWDDVEKPSSDLGAWPTGATNFSTAKQDDYGYYLDI
KMKDETASKISLLINNTSGDNITGDKTIERISTKMNEAWFDENYQLSLYQPLKEGYIRINYFRTDGNYDQ
KGLWIWGDVTDLTLGDWPNGIDFENQGKYGAYIDVKLTDLPSSIGFLLLDESKSGDDVKIQQKDYSFKDL
KNQTQIFLKDDDATIYTNPYFVNNVRATGVSHVSLTALEAAFTTLEGADKDSILEKLSVTDKNGQTVAVT
DLVLDLTSNKVRVLGDFNQENAGYTLKYGNDSFTTTMSWQLKDELYAYDGELGARVRQAGSVVDMTLWSP
SADSVAVVLYDKDDQSKVVGKLAMTKGDKGQWDLELNSQSGLGIADYRGYYYHYEITRGDQSVLVLDPYA
KSLAEWNSDLADTDPSYRIAKAAIVDPAEVGPSDLNYATIPNFNQREDAIIYEAHVRDFTADPAISDELT
AQFGTFAAFAERLSYLKELGVTHIQLLPVMSYYFVNELKNAERMSEYASSNSNYNWGYDPQSYFAFTGMY
STDPTDPMKRIEEFKNLVNEIHKQGMGVILDVVYNHTSKTFLFEDLEPNYYHFMEADGTAKSSFGGGRLG
TTHYMSRRVLVDSIKYLVDEFKVDGFRFDMMGDHDAEAIELAFTEAQKLNPNIIMLGEGWRTFTGDANQP
VQPADQDWMSSTDTVAVFSDEIRNTLKSGYPNEGQPAFITGGAKSVESVFNNIKAQPGNFLADDPGDVIQ
YIAAHDNLTLFDIIAQSIKKDPSVTENYTEIHQRQRLGNLLVLTAQGTPFIHSGQEYGRTKQFRHPDYKE
PVTEDKVPNKAHLLTNADGTPFDYPYYIHDSYDSSDAVNKFDWTKATDEALYPENTRTQAFTKGLIALRK
STDAFRLGTKEEVEQKVSLISIPGQNGIAKNDVVIAYQTIASNGDRYAVFVNADSKERSFVLSDIYKELL
KGQVLVDGERAGVEALSDLVGVELTDSSVVLAPLTATVIRLPYITTALPDTAPTAEEKPSLDFTTKERTE
ETVLPIAEEVRYDATLAKGQSYVLQEGKAGKRVLVYQDVLVDGKVVATNLLSETVVDGEARIVVKGSMEA
KDVVEKPSLSTPTAQASGQATSGASKASLPITGDRQSDLALLGLGLAGLGLTVAAQGRTKKSEE

A0A0H3MYG2-Surfome

>tr|A0A0H3MYG2|A0A0H3MYG2_STRS4 Putative surface-anchored amylopullulanase OS=Streptococcus suis (strain BM407) OX=568814 GN=SSUBM407_1919 PE=3 SV=1
MKRTGMLEKKQIFGIRKLNVGVASVGIAAALFLSGAGQLVQAEEVVLPASTSSTTLSEQDGGAVEATTTA
IEATDSMVAEATAVTETADATAPEEAPIEEGSIRLHFENVDETAPESQGLWTWGGVAEPSDGNQWPTDTA
NFSSSQVDDYGHYVDIKKSETPGTIGYLVLKNGEKITESDQKVELLVPEQNEAWIASDYSVSSYEPLKDE
NVLRINYTREDNNYEGWGVWTWGDTTEASSGWPAGAVDFKLGKYGAYVDIPLSNGLDSKLGFLLINQNNP
DLAGNKTIDLAFADRKRHSQIFLRNDDDKVYTNPYFIEEKVELDTSKATPGTKNVTVGASVKAPFNYNES
GLVSVTVTNPENAEIVKMEVDTSAIGGGLVPISTELNRVTIKATSSTAPGTYSLPVKVYDKDNGYYETKL
DVTITERIKADGEKDWDEQVIYFMMTDRFYNGDTGNDKLVEGTASNPRGLYQGGDFKGVTAKLDYLKELG
VDSIWLTPIVENIPQNVGSVKDGEYYAYHGYWASNFEKLNPHLGSLADFHELIDAAAEKGINIIVDVVLN
HAGYGTEETFAGMVRTKEEDKQGDDQLGSLSGLPDFKTEEAAVRNQLVAWQASWLERSTTAKGNSIYAFR
VDTVKHVDDTTWQHFKNELVDRDPDFHLIGETWGANYKDTKGDLGIGTMDSLLDFGFKDIAKYLVNGQLK
AAGKELEERSKVLTSAASLGQFLGSHDEDGFLYSLGGAEKEGNLDKLKLAASLLITAKGQPVIYYGEEVG
QSGQNNWPVYDNRYDFDWSKVETSDITDHYQKLLAFRNANSTLLSRGDTSTLAGNDSQGWLISKRSYQDQ
AAYLVFSTNTENKEMALEVSGKDVVVTDAYTGKSYQAIEKDGKWVVQIELPTIGQGGTMLLQTEAGDIVN
ASVQGATEEPIEAGYFRVHFKTLPSDNLSSLGLWTWDDVEKPSSDLGAWPTGATNFSTAKQDDYGYYLDI
KMKDETASKISLLINNTSGDNITGDKTIERISTKMNEAWFDENYQLSLYQPLKEGYIRINYFRTDGNYDQ
KGLWIWGDVTDLTLGDWPNGIDFENQGKYGAYIDVKLTDLPSSIGFLLLDESKSGDDVKIQQKDYSFKDL
KNQTQIFLKDDDATIYTNPYFVNNVRATGVSHVSLTALEAAFTTLEGADKDSILEKLSVTDKNGQTVAVT

DLVLDLTSNKVRVLGDFNQENAGYTLKYGNDSFTTTMSWQLKDELYAYDGELGARVRQAGSVVDMTLWSP
SADSVAVVLYDKDDQSKVVGKLAMTKGDKGQWDLELNSQSGLGIADYRGYYYHYEITRGDQSVLVLDPYA
KSLAEWNSDLADTDPSYRIAKAAIVDPAEVGPSDLNYATIPNFNQREDAIIYEAHVRDFTADPAISDELT
AQFGTFAAFAERLSYLKELGVTHIQLLPVMSYYFVNELKNAERMSEYASSNSNYNWGYDPQSYFAFTGMY
STDPTDPMKRIEEFKNLVNEIHKQGMGVILDVVYNHTSKTFLFEDLEPNYYHFMEADGTAKSSFGGGRLG
TTHYMSRRVLVDSIKYLVDEFKVDGFRFDMMGDHDAEAIELAFTEAQKLNPNIIMLGEGWRTFTGDANQP
VQPADQDWMSSTDTVAVFSDEIRNTLKSGYPNEGQPAFITGGAKSVESVFNNIKAQPGNFLADDPGDVIQ
YIAAHDNLTLFDIIAQSIKKDPSVTENYTEIHQRQRLGNLLVLTAQGTPFIHSGQEYGRTKQFRHPDYKE
PVTEDKVPNKAHLLTNADGTPFDYPYYIHDSYDSSDAVNKFDWTKATDEALYPENTRTQAFTKGLIALRK
STDAFRLGTKEEVEQKVSLISIPGQNGIAKNDVVIAYQTIASNGDRYAVFVNADSKERSFVLSDIYKELL
KGQVLVDGERAGVEALSDLVGVELTDSSVVLAPLTATVIRLPYITTALPDTAPTAEEKPSLDFTTKERTE
ETVLPIAEEVRYDATLAKGQSYVLQEGKAGKRVLVYQDVLVDGKVVATNLLSETVVDGEARIVVKGSMEA
KDVVEKPSLSTPTAQASGQATSGASKASLPITGDRQSDLALLGLGLAGLGLTVAAQGRTKKSEE

A0A0H3MXR4-Exoproteome

>tr|A0A0H3MXR4|A0A0H3MXR4_STRS4 2',3'-cyclic-nucleotide 2'-phosphodiesterase/3'-nucleotidase OS=Streptococcus suis (strain BM407) OX=568814 GN=SSUBM407_1949 PE=3 SV=1
MNFRFSKCAVALTLALLAASNPKLAQAEEILNTTPASSTEASQAVPVESDTTEEADNTESPVPATTEAEN
PSSSETAETSDPTSETTDTTTSEARTVTPAATETSQPVEGQTVDVRILATTDLHTNLVNYDYYQDKPVET
LGLAKTAVLIEEAKKENPNVVLVDNGDTIQGTPLGNYKSIVDPIEEGEQHPMYAALETLGFDVGTLGNHE
FNYGLAYLEKVIRTANMPLVNANVLDPTTKDFLYTPYTIVKKTFTDTEGKKVTLNVGVTGIVPPQILNWD
KAYLEGKVIVRDAVEAVRDIIPTMRENGADIVLVLSHSGIGDDQYEVGEENVGYQIASLSGVDAVITGHS
HAEFPGTAEKPSFYAKYSGVDDTNGKINGTPVTMAGKYGDHLGVIDLNLVFKDGKWTTTSSKAAIRKIDT
KSSVADGRIIDLAKEAHNETIKYVRQQVGETTAPINSFFALVQDDPSVQIVNNAQIWYAKQQLAGTSEAN
LPILSAAAPFKAGTRGDASAYTDIPAGPIAIKNVADLYLYDNVVAILKVNGAQLKEWLEMSAGQFNQVDL
SSTEPQNLVNTDFRTYNFDVIDGVTYQYDITQPNKYDRDGKIVNETASRVRNLQYNGQDVTADQEFIVVT
NNYRANGTFPGVREASINRLLNLENRQAIINYIIAEKVINPTADNNWTFTDSIKGLDLRFLTADRAKSLV
TDQECIVYLQASTASEGFGEFKFVYTESKVVTPDEQQSDQGNTGQDIVLESGQRITLPAVNPPAPAPQHK
LASPHSQASTKTLPATGEATSMLSLLGLTLIGFVGAWTKKKEH

A0A0H3MXR4-Surfome

>tr|A0A0H3MXR4|A0A0H3MXR4_STRS4 2',3'-cyclic-nucleotide 2'-phosphodiesterase/3'-nucleotidase OS=Streptococcus suis (strain BM407) OX=568814 GN=SSUBM407_1949 PE=3 SV=1
MNFRFSKCAVALTLALLAASNPKLAQAEEILNTTPASSTEASQAVPVESDTTEEADNTESPVPATTEAEN
PSSSETAETSDPTSETTDTTTSEARTVTPAATETSQPVEGQTVDVRILATTDLHTNLVNYDYYQDKPVET
LGLAKTAVLIEEAKKENPNVVLVDNGDTIQGTPLGNYKSIVDPIEEGEQHPMYAALETLGFDVGTLGNHE
FNYGLAYLEKVIRTANMPLVNANVLDPTTKDFLYTPYTIVKKTFTDTEGKKVTLNVGVTGIVPPQILNWD
KAYLEGKVIVRDAVEAVRDIIPTMRENGADIVLVLSHSGIGDDQYEVGEENVGYQIASLSGVDAVITGHS
HAEFPGTAEKPSFYAKYSGVDDTNGKINGTPVTMAGKYGDHLGVIDLNLVFKDGKWTTTSSKAAIRKIDT
KSSVADGRIIDLAKEAHNETIKYVRQQVGETTAPINSFFALVQDDPSVQIVNNAQIWYAKQQLAGTSEAN
LPILSAAAPFKAGTRGDASAYTDIPAGPIAIKNVADLYLYDNVVAILKVNGAQLKEWLEMSAGQFNQVDL
SSTEPQNLVNTDFRTYNFDVIDGVTYQYDITQPNKYDRDGKIVNETASRVRNLQYNGQDVTADQEFIVVT
NNYRANGTFPGVREASINRLLNLENRQAIINYIIAEKVINPTADNNWTFTDSIKGLDLRFLTADRAKSLV
TDQECIVYLQASTASEGFGEFKFVYTESKVVTPDEQQSDQGNTGQDIVLESGQRITLPAVNPPAPAPQHK
LASPHSQASTKTLPATGEATSMLSLLGLTLIGFVGAWTKKKEH

A0A0H3MV01-Exoproteome

>tr|A0A0H3MV01|A0A0H3MV01_STRS4 Putative IgA-specific zinc metalloproteinase OS=Streptococcus suis (strain BM407) OX=568814 GN=zmpC PE=4 SV=1
MKRSLGEKRQRFGFRKMSVGLVSAAVTSLFFVSSVATAPTASAQSINYSYVTEQELTDGEKELIIRDLPG
LAQETDVNYYLIYRPATGTTSTPSTSTSQVLPNTGSVETELLVAGGVSLLLLAVRFGKKGKKELAGVILL
TATGASFFGPTSSALTSQILARYNHAIEISAGQALPAPAEIDGYVYVGYLKDSKAIEQTTSEEEKTAEFP
ASEGIRTETIVNKTEAIPFEIQTVENPQLSAGTERVVQEGQDGERIVTIKQVHSKGQIISEEEISSTVTK
TAVPKIVEVGTKQATDDIVTEVPDTEPSYENSTNSLTEETVTHTEVFAFDVQEIYDVNLAEGSREVEQKG
QDGVRTIETRNYYADGVLIKSEQVSDVVTKEPVTEVVRVGTKTTDVIGSETIVTTEELPFETTVTETEEL
YVGEEKFITEGKVGSKEVTTTYQTINGVSQPNPTVTEKVLEEPTTKEVLKGTKPIEGTEIETNQVEITFE
TEYVDDPTLLEGKTKVVTAGVNGSKTVTTTYQTIKGVRQENSTVTEEITKQPVKQVIARGTKVEKVPQVI
ITDLVENDDAKSATISYKLTDETANFQRAVALLYDNTGALVQEQTITDPNGQLTLENLDFYTDYTVKTKI
FYTMAEQEQSSEQEAILESMRKFDLVYKKIEIKDIDAVTVYRRKNGSYIGQEFLEELPASTDELFIKVTS
DRFKEVYLPVSSIEETTLNGKAVFKLVSSFDELVQDKDAQYVANREFYIPKMATDANTYTSFKALLDAMK
ANPSGTFKLGAHLDASEVPVGDVASYVTNFSGTLDGLNDGYAFSISNLKAPLFFNLGGKVQNLDIKNASL
NTSSKNPLATIAINANGATITNVAVEASIKGPQNVSGLVHSATNTTIKGVSFKGSIEVTGTNASLTGGIL
GNGTMASVGNAKVDATITLPGTENQVAGGIVGRTMLVYDVPGSVYNSYAAGSIVTTESAAIVGGIAGANQ
VTGAYAPHSGNVNNVVSDMTGTKSIIGQPANPTGKIKDGFTTTSDSLTNVTVITDEEAQAKVEAMAIQAT
IDDSVPLNPNHYSVDYLTLDKAQADHETAYYNMEKILPFYNKELLVYYGNKIATDDKLNKVRLLDVVPMK
DNAVVADVYAEKANINKIMLHYADGTVDYKTVSYLEDFKNNHVVEYTISGTDLIYTPESFLNDRSALVND
LVSSLSSVVLDSDAMKAVINYPTTLNADTQTGTAKDFYFGESYDQVMTNLESNVRKILVASLNGQGQASE
DYIKEKIINNKAAFVLGLTYLNRWYDINFGEMNTKDLTIFEPDFFGNDAASALDMILAIGNGGYDVLRAH
NNVTTFASIIGKQNNQTKLFDMLEDYRQLFLPEMTNNEWFKQTTKAYVVEGKSLIPEVAAKQETTDTYSK
YNVGAYSKIVNDTVSNPTWKYNHMLLPLLTLPQENIFIITNMNTIAIGSYEHYVDDYSTVENRDKVRQMV
DLAAERQRDNADFWYKILDETNRDKLFRSVLNNEGYVMYGKDGTKSYRNLTADVDAIQDFYGPINKWYRE
HPSIKTAFADGSETYYITYDMLTDYGTALYTHEMVHNQDGDIYLKGYGRRIGQGMEVYAQGLLQNVFNVT
EMNLGFNAVYNSDDANRVHVGDPVARFNSEADFNEYYHNQFDVLYLLDYLEGTNILAQSDANKKAWLRKI
ENYYVQNNGVDTHAGNSARALTDAEVASLKSFNDLIDQSIIVQRQYVNNPTNTSKKWDRNSYVSVPMFAA
NFSALSNSNGSPGDIMFRRMAFELIAAKGYTDGFVPYASGQLSDLAMEKGSIIYDTWNKKNTGLITDDHV
LEYVFQGQYTSWTEFKKAMFTERLEKAATGQLKPFTMQYELGVADSTKEVTITSFEQLQNLMKEAMEADI
QANSLNLNNSRVHALKVKVYQALMNSTNDFRTSIFN

A0A0H3MV01-Surfome

>tr|A0A0H3MV01|A0A0H3MV01_STRS4 Putative IgA-specific zinc metalloproteinase OS=Streptococcus suis (strain BM407) OX=568814 GN=zmpC PE=4 SV=1
MKRSLGEKRQRFGFRKMSVGLVSAAVTSLFFVSSVATAPTASAQSINYSYVTEQELTDGEKELIIRDLPG
LAQETDVNYYLIYRPATGTTSTPSTSTSQVLPNTGSVETELLVAGGVSLLLLAVRFGKKGKKELAGVILL
TATGASFFGPTSSALTSQILARYNHAIEISAGQALPAPAEIDGYVYVGYLKDSKAIEQTTSEEEKTAEFP
ASEGIRTETIVNKTEAIPFEIQTVENPQLSAGTERVVQEGQDGERIVTIKQVHSKGQIISEEEISSTVTK
TAVPKIVEVGTKQATDDIVTEVPDTEPSYENSTNSLTEETVTHTEVFAFDVQEIYDVNLAEGSREVEQKG
QDGVRTIETRNYYADGVLIKSEQVSDVVTKEPVTEVVRVGTKTTDVIGSETIVTTEELPFETTVTETEEL
YVGEEKFITEGKVGSKEVTTTYQTINGVSQPNPTVTEKVLEEPTTKEVLKGTKPIEGTEIETNQVEITFE
TEYVDDPTLLEGKTKVVTAGVNGSKTVTTTYQTIKGVRQENSTVTEEITKQPVKQVIARGTKVEKVPQVI
ITDLVENDDAKSATISYKLTDETANFQRAVALLYDNTGALVQEQTITDPNGQLTLENLDFYTDYTVKTKI
FYTMAEQEQSSEQEAILESMRKFDLVYKKIEIKDIDAVTVYRRKNGSYIGQEFLEELPASTDELFIKVTS
DRFKEVYLPVSSIEETTLNGKAVFKLVSSFDELVQDKDAQYVANREFYIPKMATDANTYTSFKALLDAMK
ANPSGTFKLGAHLDASEVPVGDVASYVTNFSGTLDGLNDGYAFSISNLKAPLFFNLGGKVQNLDIKNASL
NTSSKNPLATIAINANGATITNVAVEASIKGPQNVSGLVHSATNTTIKGVSFKGSIEVTGTNASLTGGIL
GNGTMASVGNAKVDATITLPGTENQVAGGIVGRTMLVYDVPGSVYNSYAAGSIVTTESAAIVGGIAGANQ
VTGAYAPHSGNVNNVVSDMTGTKSIIGQPANPTGKIKDGFTTTSDSLTNVTVITDEEAQAKVEAMAIQAT
IDDSVPLNPNHYSVDYLTLDKAQADHETAYYNMEKILPFYNKELLVYYGNKIATDDKLNKVRLLDVVPMK
DNAVVADVYAEKANINKIMLHYADGTVDYKTVSYLEDFKNNHVVEYTISGTDLIYTPESFLNDRSALVND
LVSSLSSVVLDSDAMKAVINYPTTLNADTQTGTAKDFYFGESYDQVMTNLESNVRKILVASLNGQGQASE

DYIKEKIINNKAAFVLGLTYLNRWYDINFGEMNTKDLTIFEPDFFGNDAASALDMILAIGNGGYDVLRAH
NNVTTFASIIGKQNNQTKLFDMLEDYRQLFLPEMTNNEWFKQTTKAYVVEGKSLIPEVAAKQETTDTYSK
YNVGAYSKIVNDTVSNPTWKYNHMLLPLLTLPQENIFIITNMNTIAIGSYEHYVDDYSTVENRDKVRQMV
DLAAERQRDNADFWYKILDETNRDKLFRSVLNNEGYVMYGKDGTKSYRNLTADVDAIQDFYGPINKWYRE
HPSIKTAFADGSETYYITYDMLTDYGTALYTHEMVHNQDGDIYLKGYGRRIGQGMEVYAQGLLQNVFNVT
EMNLGFNAVYNSDDANRVHVGDPVARFNSEADFNEYYHNQFDVLYLLDYLEGTNILAQSDANKKAWLRKI
ENYYVQNNGVDTHAGNSARALTDAEVASLKSFNDLIDQSIIVQRQYVNNPTNTSKKWDRNSYVSVPMFAA
NFSALSNSNGSPGDIMFRRMAFELIAAKGYTDGFVPYASGQLSDLAMEKGSIIYDTWNKKNTGLITDDHV
LEYVFQGQYTSWTEFKKAMFTERLEKAATGQLKPFTMQYELGVADSTKEVTITSFEQLQNLMKEAMEADI
QANSLNLNNSRVHALKVKVYQALMNSTNDFRTSIFN
